# Municipal solid waste management: Identification and analysis of technology selection criteria using Fuzzy Delphi and Fuzzy DEMATEL technique

**DOI:** 10.1016/j.heliyon.2023.e23236

**Published:** 2023-12-05

**Authors:** Mehedi Hasan Shanta, Imtiaz Ahmed Choudhury, Sheak Salman

**Affiliations:** Department of Mechanical and Production Engineering, Ahsanullah University of Science and Technology, Dhaka, 1208, Bangladesh

**Keywords:** Municipal solid waste management (MSWM), Technology selection, Developing countries, Multi criteria decision making (MCDM), Fuzzy set theory, Fuzzy Delphi method, Fuzzy DEMATEL

## Abstract

Municipal solid waste management (MSWM) poses a considerable challenge to developing countries like Bangladesh because of the rising waste generation rates and lack of effective management practices such as illegal open dumping and informal waste collection. One of the crucial factors in the successful management of MSW is to select the appropriate technology which is a complex multi-criteria and laborious process. Despite the global emphasis on the importance of MSWM in the literature, there is a lack of studies conducted in developing countries that effectively identify and analyze the critical performance criteria for appropriate technology selection. This research aims to address this shortcoming by identifying, and prioritizing the selection criteria and finally investigating the inter-relationship between them and the degree to which they affect or are affected by one another. First, a thorough literature review and expert consultation were employed to determine a set of 21 key criteria using the Fuzzy Delphi method (FDM). Later, taking into account the imprecise and subjective nature of the DEMATEL method on human judgements, the Fuzzy DEMATEL technique was employed to investigate the cause-effect relationships among the identified criteria. The findings of the study demonstrated that 14 criteria were categorized as causal elements that have the most significant influence on the MSWM technology selection process and 7 criteria were categorized as effect. The selection of MSWM technology demands greater consideration of the top three ranked criteria, namely **T**_**4**-_ Access to Technology (AT), **T**_**8**-_ Feasibility (F), and the **Ec**_**6**_-Infrastructure requirements (IR). By identifying the pertinent criteria, structures and interrelationships, the outcome of the study can facilitate a better understanding of causal relationships among the criteria that require specific consideration from the decision-makers and allow them to select appropriate MSW management technology.

## Highlights

• Identified 21 key performance criteria for Bangladesh's MSWM technology selection.
• Developed a Cause & Effect model and ranked the criteria using the Fuzzy DEMATEL technique.
• The top-ranked cause criteria, **T**_**4**-_ Access to Technology (AT) requires the highest attention.
• Constructed a causal interactions diagram to understand the degree of causal relationships.
• Highlighted the theoretical and managerial implications.

## 1 Introduction

Municipal solid waste management (MSWM) represents a substantial challenge in various urban regions worldwide. Annually, an estimated 2.01 billion metric tonnes of municipal solid waste (MSW) are generated globally. By the year 2030, it is projected that there will be an annual generation of 2.59 billion tonnes of MSW, and by the year 2050, it will be around 3.40 billion tonnes where 33 % of the waste is not being processed in an ecologically sustainable manner. It requires urgent attention to address this issue. In underdeveloped countries where the economic resources are limited, the effect of this situation is detrimental. Geographical location and economic development affect the MSW generation in underdeveloped countries, which ranges from 0.11 to 4.54 kg per day. It is anticipated that there will be an increase of 19 % and 40 % in waste generation in the developed and developing nations, respectively. In recent years, the regions of South Asia and East Asia (Pacific) are expected to exhibit the most significant growth rates for MSW generation. More specifically, South Asia is anticipated to experience a surge in MSW generation that is more than double its current level [1]. Due to several circumstances, including urbanization, rapid population expansion, inadequate planning, and a lack of a scientific approach, issues with solid waste management (SWM) practises are particularly acute in developing countries [2].

As a developing country, the condition of waste management in Bangladesh is a matter of significant concern as it poses major risks to both human and environmental health. Bangladesh has demonstrated a noticeable disregard for environmental concerns in the field of solid waste management [3]. Ahsan et al. [4] found from their investigation that waste generation rates in different Bangladeshi cities range from 0.2 to 0.56 kg/cap/day. Bhuiyan et al. [5] stated that by 2025, trash production in Bangladesh's municipal regions is predicted to rise by 0.6 kg/cap/day. It is noteworthy that exclusively the capital city, Dhaka produces more than 5000 metric tonnes of solid waste material every day [6]. Plastic wastes carry the major portion of the total generated SW of Bangladesh. According to Islam et al. [7], Bangladesh generates about 8 % of global waste and 3000 tonnes of plastic waste daily. Although the handling of plastic waste in municipal areas is still in its early stages, Bangladesh also lacks the specific guidelines, educational resources, legislation, and policies that would enable the successful management of MSW [8]. Developing nations like Bangladesh frequently lack the necessary effective waste management technology or policies for the efficient treatment of their waste. The SWM practices in Bangladesh predominantly involve unregulated open dumping, incineration, landfilling, waste disposal in aquatic bodies, direct disposal onto farmland in rural areas, unestablished recycling firms, and informal waste collection during its initial and subsequent phases. Inadequate waste management practices are characterized by the utilization of inefficient technology, a lack of technical support, and notably insufficient financial support for the implementation of waste management techniques [9,10].

Despite the abundance of literature on the global waste generation and handling of MSW, this particular domain tends to receive the least scholarly attention in Bangladesh. Studies indicated that the absence of appropriate technology selection serves as a significant obstacle to achieve efficient SWM in Bangladesh and highlighted the selection process as multifaceted and complex. The implementation of appropriate technology selection for MSWM can help to address the problem of inefficient MSWM in Bangladesh. The management system of the SW in Bangladesh remains inadequate despite numerous initiatives by both government and non-government organizations due to the absence of appropriate technology selection. That is why, it is a big challenge to identify the appropriate MSWM technology. Though some technologies including Landfills (Landfill Gases, Landfill Leachate), Thermal techniques (Incineration, Gasification, Pyrolysis), and Biochemical conversion (Anaerobic Digestion, Composting) are practised in Bangladesh, the importance of sustainable and reliable MSWM technology selection is a must thing now to achieve an effective MSW management system [1]. Since there is limited literature available on Bangladesh's MSWM, related studies from regions with similar socio-economic conditions provided an overview of the method used to resolve these problems.

In a case study from India, the authors proposed a three-stage evaluation method that uses three complimentary MCDM techniques including the Fuzzy Delphi method (FDM), FAHP, and FTOPSIS to make the SWM decision-making fast, robust, and reliable. They evaluated six different SWM technology considering 12 relevant criteria based on expert's opinions [11]. Similarly, a study aimed to determine the best disposal technology using three MCDM methods: TOPSIS, PROMETHEE, and Fuzzy TOPSIS. They evaluated 18 criteria, consisting of 8 cost criteria and 10 benefit criteria [12]. The combination of Fuzzy logic and the DEMATEL technique is used in a study to provide an effective solution to assist an expert group in making decisions related to MSW management. The research concentrates on the Metro Manila region and employs unbiased benchmarks that encompass all 17 crucial criteria associated with municipal solid waste (MSW) management and a Cause & Effect model was established [13]. Another study related to SWM used the Fuzzy Delphi Method (FDM) to screen and select criteria for landfill site selection where the researchers identified 21 criteria for MSW landfill site selection and then applied the Fuzzy AHP and DEMATEL technique to understand the interrelationships among the identified criteria for landfill site selection [2].

According to our knowledge, very limited research on MSWM has been conducted in Bangladesh, and none of those studies have attempted to identify the significant performance criteria and the interrelationships among the criteria. Other than these, the causal interactions among the criteria have not been studied yet as well. Therefore, it is necessary to analyze to figure out the interdependencies among the criteria. Moreover, it appears that several studies have neglected to consider the provision of inaccurate data and the degree of potential ambiguity in the decision-making process, other than lacking any reasonable explanation. To investigate and deal with these challenges, a set of research questions (*RQ*s) have been formulated below.

RQ1 What are the significant performance criteria influencing municipal solid waste management technology selection in Bangladesh?

RQ2 What are the cause-and-effect relationships among the identified criteria?

RQ3 What is the level of causal relationships among the identified criteria?

RQ4 What are the implications of those performance criteria?

This paper makes three contributions to the existing literature in response to the above-mentioned *RQ*s. Firstly, a thorough literature review and consultation with a panel of experts enabled the identification of 21 significant performance criteria for the selection of MSWM technology through the Fuzzy Delphi method (FDM). Secondly, a decision-making trial and evaluation laboratory (DEMATEL) method is considered for the benefits of this technique, which is beneficial for complex systems analysis and revealing the causal relationships among the criteria. Since FDM is well-known for its ability to effectively remove uncertainty that frequently results from a mutual understanding of specialised viewpoints, it was applied in the first stage of collective decision-making. The Fuzzy DEMATEL technique was implemented in the following stage in light of its widely accepted recommendation to handle flexible fuzziness situations in order to evaluate the importance of the identified criteria and ascertain the cause and effect criteria. Finally, the causal interactions among the identified criteria mean the degree of causal relationships was determined. The findings of this study will help the decision-makers and policymakers regarding a robust decision-making process and will keep contributing to more sustainable and responsible waste management practices in the future. The identified cause and effect criteria will help municipal professionals to decide which MSWM technology needs to be eliminated and which technology should be adopted for the municipalities.

This study is organized as follows: Section 1 introduces the background and motivation. The literature review is provided in Section 2 whereas Section 3 describes the methodological approach. Section 4 presents the results and analysis; Section 5 discusses the key findings of this study. Finally, Section 6 presents the Conclusions, implications and recommendations for future research. Table 1 below shows the abbreviations used in this paper and their meanings.

**Table 1:** Abbreviation used in this paper.

Abbreviation	Description
MSWM	Municipal solid waste management
SWM	Solid waste management
SW	Solid wastes
MCDM	Multi criteria decision making
FDM	Fuzzy Delphi method
DEMATEL	Decision-making trial and evaluation laboratory
ISM	Interpretive structural modeling
AHP	Analytic hierarchical process
TOPSIS	Technique for order performance by similarity to ideal solution
PROMETHEE	Preference Ranking Organization Method for Enrichment Evaluation
VIKOR	Vise Kriterijumska Optimizacjja I Kompro- misno Resenje
SBWM	Stratified best-worst method
CPT	Cumulative prospect theory
TFN	Triangular fuzzy numbers

## 2 Literature review

### 2.1 Municipal solid waste management (MSWM)

Municipal solid waste management (MSWM) is now a global concern due to the vast quantity of 2.01 billion metric tonnes of solid waste being produced yearly worldwide. Predictions indicate that by the year 2030 and 2050, the yearly generation of MSW would rise to 2.59 billion tonnes and 3.40 billion tonnes, with the growth rate being considerably greater in developing and poor countries [1,14]. The anticipated amount of MSW generation in Asia is projected to increase substantially to 1.8 million tonnes per day by the year 2025, higher than the present rate of 1 million tonnes per day [15]. Ahmed et al. [6] stated that studies from a number of cities across various countries, namely China, India, Malaysia, Thailand, and Bangladesh, have reported significant waste management issues due to population growth, industrialization, and urban development. According to research, South-East Asia produced 70 million tonnes of SW each day whereas Bangladesh produced 16,384 tonnes per day [16].

In Bangladesh, the generation rate of waste varies throughout the cities ranging from 0.25 to 0.70kg/capita/day, where the city, Chittagong (0.56 kg/capita/day) and Dhaka, the capital city (0.70kg/capita/day) producing the largest amount of waste. The residential sector is accountable for approximately 78 % of the overall waste production, while the industry sector contributes 20 %, and the other sectors generate the remainder [17]. The SW generation per person will exceed 0.75 kg/capita/day by 2025 and the overall amount of waste generation will reach 21.07 million tonnes per year. The amount of waste produced in Bangladesh increased to 52,00,000 tonnes in 2015, with an annual growth rate of 1,34,300 tonnes [15]. The municipal areas exhibit variance in the average per capita production of MSW, ranging from 0.2 to 0.56 kg/cap/day [18]. In the urban regions of Bangladesh, the production of waste will increase by 0.6 kg/cap/day, resulting in a total waste volume generation of 57,718 tonnes per day by the year 2025 [4]. Using the data from a case study on plastic consumption and waste management in Bangladesh, a World Bank team showed the present conditions and made recommendations for a multisectoral action plan for the long-term management of plastic [19]. According to simulated results from a study, by the year 2050, the MSW generations will increase from 168,000 tonnes in 2020 to 1.2 million tonnes in 2050 and the per capita generation of MSW is also predicted to rise from 0.117 tonnes to 0.561 tonnes during this period [20]. Dhaka, the capital city, presents the same scenario where waste management has proven to be a major challenge, as the daily amount of generated waste exceeds 4124 tonnes, of which nearly 40 % remains uncollected [21]. Even so, a number of studies have shown that Dhaka is the city that generates the most MSW (roughly 70 % of the country's total MSW) [22].

Since improper disposal of SW directly threatens human health and pollutes the environment, MSWM for developing nations like Bangladesh is the biggest problem. Hence, to achieve sustainable environmental management, sustainable SWM is the primary criterion. According to studies, waste management, particularly MSW management, is considered to be an issue of lower priority in Bangladesh which is a result of the absence of motivation and awareness among residents, insufficient implementation of policies, and financial limitations. Despite the resource limitations, the government of Bangladesh continues to be unwavering in its determination to adopt technological advances to efficiently handle waste [23].

### 2.2 MSWM technology practices in Bangladesh

Early waste management practices in Bangladesh involved uncontrolled and unrestricted open dumping and burning, as well as the disposal of waste into aquatic bodies, landfills, and direct disposal into the farmland of rural areas. As time progressed, the country evolved its waste management practices from the traditional approach to the modern approach. The current approach views waste as a resource, leading to a shift from waste management to resource management. However, a significant portion of waste, ranging from 40 % to 60 %, remains uncollected and is disposed of in a less safe manner, primarily due to a lack of awareness, motivation, financial resources, and technological advancements [4]. Insufficient collection, inadequate coverage of transport, the employment of unsuitable processing technology and treatment are responsible for the poor management of MSW which leads to environmental pollution, significant health risks, and environmental hazards [24].

The methods and techniques employed by the municipal officials of Bangladesh are traditional and comparable to those used in other developing nations which are manual and highly labour-intensive. It is common for individuals to separate valuable waste materials to sell them to vendors. Meanwhile, pickers are responsible for retrieving recyclable waste products from dumpsites, community bins, and roadsides. Incineration technology is not widely utilized due to the high financial burden associated with it, thereby leading to open dumping as the preferred landfilling method [3]. The sustainable and effective management of waste can yield fuel and electricity through the processes of composting, anaerobic digestion, and thermos chemical processing. Each of these waste treatment methods requires unique cost patterns and by reducing noxious gases, global warming, and carbon footprint, sustainable waste management can result in cost savings, as well as benefits for public and environmental health [23].

The existing MSW management practices in Bangladesh are entirely reliant on unregulated open disposal, unestablished recycling firms, and informal waste collection. An investigation highlighted various MSW management technologies, such as Landfills (Landfill Gases, Landfill Leachate), Thermal techniques (Incineration, Gasification, Pyrolysis), and Biochemical conversion (Anaerobic Digestion, Composting) and indicated the importance of proper SWM technology selection to achieve an effective MSW management system. The determination of a suitable approach for the management and treatment of MSW - including techniques such as landfill dumping, thermal treatment, and chemical processes – depends on the composition of the waste, the available technological resources, and the socioeconomic structures of the country. It is of utmost importance for developing countries to carefully choose appropriate technologies for MSW management. Bangladesh has received the least attention, even though there is a wealth of literature on the production and management of MSW all over the world [1]. Developed countries are actively pursuing the safe and efficient management of waste through the implementation of contemporary waste treatment methods, such as Anaerobic Digestion (AD), incineration, pyrolysis, gasification, and recycling to generate energy from waste [25]. Conversely, Bangladesh is less familiar with these standard techniques, lacking relevant knowledge, and failing to implement most modern waste management technologies, as documented in research findings [17].

### 2.3 Established performance criteria for SWM technology selection

The relevant works from countries with comparable socio-economic backgrounds were analyzed to establish a framework for determining the criteria for selecting an appropriate Solid Waste Management (SWM) technology. A study from India identified 12 important criteria including Net Economic cost (NEC), Technical Reliability (TR), Environmental Feasibility (EF), etc. for evaluating MSW management technology for Mumbai city [11]. Another study from Turkey aims to determine the best solid waste disposal technique where 18 criteria have been evaluated. The study considered all available solid waste disposal technologies to provide insight into potential solutions for the future [12]. A further investigation explored the utilization of MCDM and Life cycle assessment (LCA) methodologies in assessing waste management systems regarding environmental, social, and economic sustainability criteria. The authors have identified the primary and secondary criteria that are often employed in MCDM research to select waste management technologies and emphasize that waste management technology selection should consider a range of criteria to ensure that the chosen technology is technically reliable, environmentally feasible, economically viable, and socially acceptable. Overall, this literature presents valuable insights into the key criteria that should be taken into account when selecting solid waste management technology [26].

Thus, commonly employed aspects and criteria for selecting solid waste management technologies in different countries were identified from related literature which require careful consideration and screening when assessing their applicability in Bangladesh [12,[27], [28], [29], [30], [31], [32], [33], [34], [35]]. The identified criteria have been organized in Table 2 where 22 criteria were from relevant literature and the remaining 4 were suggested by the experts involved in the Fuzzy Delphi method (FDM), described in section 3.1.

**Table 2:** Established performance criteria for SWM technology selection.

Aspects	Code	Criteria	References
Technical	**C**_**1**_	Technical Reliability (TR)	[11,26,29]
	**C**_**2**_	Energy Recovery (ER)	[11,26,35]
	**C**_**3**_	Treatment Effectiveness (TE)	[11,13,36]
	**C**_**4**_	Access to Technology (AT)	[1,26,29]
	**C**_**5**_	Power generation rate (PGR)	[26,36,37]
	**C**_**6**_	Expert Personnel Requirement (EPR)	Experts suggested
	**C**_**7**_	Quality and Quantity of Labor (QL)	Experts suggested
	**C**_**8**_	Efficiency (E)	[10,26,30]
	**C**_**9**_	Feasibility (F)	[26,32,33]
Environmental	**C**_**10**_	Environmental Feasibility (EF)	[11,26,34]
	**C**_**11**_	Air Pollution Control (APC)	[11,26,36]
	**C**_**12**_	Emission Control Levels (ECL)	[27,28,30]
	**C**_**13**_	Water Pollution (WP)	[12,26,38]
	**C**_**14**_	Global Warming (GW)	[11,12,26]
	**C**_**15**_	Soil Pollution (SP)	[21,26,39]
	**C**_**16**_	SO_X_ and NO_X_ emissions (SNE)	[26,27,40]
Economic	**C**_**17**_	Net Economic Cost/Net Cost per ton of waste (NEC)	[11,12,26]
	**C**_**18**_	Initial investment cost (IIC)	Experts suggested
	**C**_**19**_	Operational Cost (OC)	[11,26,37]
	**C**_**20**_	Revenues (R)	[3,26,41]
	**C**_**21**_	Transportation Costs (TC)	[4,31,32]
	**C**_**22**_	Maintenance Cost (MC)	[11,26,32]
	**C**_**23**_	Infrastructure Requirements (IR)	Experts suggested
Social	**C**_**24**_	Public Acceptance (PA)	[11,33,42]
	**C**_**25**_	Political Support (PS)	[6,34,43]
	**C**_**26**_	Awareness (A)	[8,26,31]

Note: C_1,_ C_2,_ C_3,. …,_ C_26_ indicates the criteria.

### 2.4 Existing methods used in MSWM studies

MCDM methodologies have attained widespread recognition due to their ability to analyze complex real-world situations. These techniques were developed for group decision-making and there are numerous efficient MCDM techniques for analyzing issues with group decision-making [44]. The utilization of MCDM approaches is essential in MSWM as it can solve complex decision-making problems and ensure sustainable and robust decision-making. Though several MCDM techniques are utilized for different objectives, Decision-making trial and evaluation laboratory (DEMATEL), Interpretive structural modeling (ISM), Analytic hierarchical process (AHP), Vise Kriterijumska Optimizacjja I Kompro-misno Resenje (VIKOR), Technique for order performance by similarity to ideal solution (TOPSIS), are the most frequently utilized techniques [45]. Based on the research conducted, when the quantity of variables is increased, the DEMATEL technique exhibits superior performance in assessing pairwise comparisons, while the AHP technique has limitations. Quantitative findings from the TOPSIS approach require the combination of any other method with it and despite being good at the ranking process, VIKOR performs poorly with a larger number of variables [46].

To overcome these issues, the DEMATEL technique is applied to address the significance and causal relationships among the criteria, as well as to identify the influential criteria in the selection of MSWM technology and to construct the causal relationship diagram [2]. The utilization of DEMATEL presents numerous benefits as it enables the identification of interrelationships among factors and facilitates the prioritization of criteria where the prioritization is achieved by evaluating the relationships and the level of their effects on each other [47]. Considering these benefits, DEMATEL is employed to identify the effect and cause criteria and to obtain the linguistics model which is parameterized with triangular fuzzy numbers. Furthermore, the utilization of Fuzzy DEMATEL methodology is integrated to generate a higher degree of precision in the analysis which represents an extended approach created to tackle complex elements in a fuzzy domain [13,46]. This analytical method offers comprehensive insight by providing a clearer understanding of the overall structure.

Although the utilization of MCDM techniques in MSWM holds great advantages in facilitating sound decision-making, the relevant literature on the implementation of MCDM in solid waste management in Bangladesh is very limited. This requires critical analysis of the literature from countries with similar socio-economic conditions like Bangladesh.

To address the problems with solid waste disposal in Dhaka, Bangladesh, an article suggested the use of appropriate waste-to-energy conversion technology. It compared three alternatives and 9 criteria, focusing on technological, environmental, and financial aspects, and finally provided insight for sound decision-making [33]. A study from India suggested a three-stage evaluation method using three complementary MCDM techniques for fast, robust, and reliable decision-making. The initial stage uses the Fuzzy Delphi method (FDM) to establish criteria for MSW management technology, followed by the Fuzzy AHP to determine the decision-maker's opinions on each criterion's relative importance and finally, the Fuzzy TOPSIS methodology is used to choose the most suitable MSW treatment and disposal techniques [11]. Arıkan et al. [12] evaluated solid waste disposal techniques in Turkey using TOPSIS, PROMETHEE, and Fuzzy TOPSIS, considering 18 criteria. It aims to identify future solutions and settle experts' opinions by determining the geometric average of survey results. Coban et al. [38] investigated various solid waste disposal techniques by utilizing the TOPSIS and PROMETHEE methods to determine the most appropriate and feasible ones. The method for choosing a sustainable waste disposal system using MCDM methodologies is proposed in a different study where it combines the stratified MCDM methodology with the best-worst method to eliminate the ambiguity of future criterion weightings [32]. Mascarenhas et al. [28] aimed to support the decision-making process for SW treatment technologies in Kisumu, Kenya by analyzing multiple socioeconomic and environmental parameters of different treatment technologies. The authors employed a multi-criteria analysis approach to evaluate and compare the anaerobic digestion, sanitary landfill, bioreactor landfill, and incineration options, based on multiple criteria. Another study utilized FDM to identify 21 relevant criteria for MSW landfill site selection through literature review, expert opinions, and decision-maker's knowledge. Then the Fuzzy-AHP approach was employed to evaluate and assign significance to the criteria, while the DEMATEL method was utilized to identify the interdependencies and significant criteria [2]. The Metro Manila region was the focus of a study that employed unbiased benchmarks and included 17 crucial criteria associated with MSW management. The study used the combination of fuzzy logic and DEMATEL to provide an effective solution to assist an expert group in making decisions. Finally, a Cause & Effect model was developed to get valuable insight into MSWM [13]. Similarly, a paper from Shanghai presents an MCDM method for selecting suitable plastic waste treatment technology using cumulative prospect theory (CPT) and the Fuzzy DEMATEL technique. The model studies seven alternatives including landfill, recycling, pyrolysis, incineration, and a combination of the alternatives [37].

Table 3 summarizes the MCDM techniques used to solve different MSWM issues in different countries. However, no research has been reported yet that identifies the key performance criteria for MSWM technology selection in the context of Bangladesh. Furthermore, the interrelationships among the criteria are also not determined, which is another factor that is crucial for making sound decisions in MSWM. In this research, the integration of the Fuzzy Delphi method (FDM) and the Fuzzy DEMATEL technique has been incorporated to address these gaps.

**Table 3:** Existing studies related to the MCDM approaches in MSW management.

Literature(s)	Application area	Case study	Method used
[32]	Waste disposal technology selection	Iran	BWM
[51]	Waste treatment technology selection	Canary archipelago	Fuzzy TOPSIS
[11]	Waste treatment technology selection	India	FAHP-TOPSIS
[12,38]	Waste treatment technology selection	Turkey	PROMOTHEE-TOPSIS
[48]	Waste treatment method selection	–	CRITIC-MULTIMOORA
[33]	Waste-to-Energy conversion technology selection	Dhaka, Bangladesh	AHP
[2]	Landfill site selection	–	Fuzzy AHP, Fuzzy DEMATEL
[13,37]	Waste treatment technology selection	Manila, China	Fuzzy DEMATEL
[34,49]	waste treatment technology selection	China, India	AHP
[50]	Dismantling Location Selection	Istanbul, Turkey	CODAS
[39]	Disposal technology selection	Africa	LCA-AHP-VIKOR
[52]	Disposal technology selection	–	Fuzzy DEMATEL
[35]	Waste collection trucks selection	Spain	SBWM

### 2.5 Research gaps and significant contributions

As mentioned in Table 3, several research projects from different countries applied different approaches to solve the concerning issues regarding MSWM. Two studies ([12,38]) used the PROMOTHEE-TOPSIS method to select solid waste disposal techniques for Turkey considering various criteria. The other studies ([34,48,49]) used the CRITIC-MULTIMOORA, AHP approaches to choose the waste treatment method, although the outcomes of these methods are biased and ambiguous. Likewise, for selecting the disposal technology, a study [39] used the LCA-AHP-VIKOR and the DEMATEL method respectively and to select the dismantling Location, CODAS is used in a study [50]. The AHP approach is used in a study to choose the waste-to-energy conversion technology comparing only three alternatives and 9 criteria for Dhaka, Bangladesh, without even considering the ambiguity in opinions and judgements [33]. Conversely, other studies ([11,13,32,35,37,51,52]) considered these issues and utilized the fuzzy-based methods, namely, Fuzzy TOPSIS, FAHP-TOPSIS, Fuzzy DEMATEL and other recently developed methods, namely, SBWM to eliminate the biases in expert's judgements.

The critical review and analysis of the literature related to MSWM and the technology selection from different countries reveal that many of these studies not only lacked a rational justification but also failed to consider the possibility of imprecise data and the level of ambiguity present in the process of making decisions. However, in Bangladesh, no extensive research has been done in SWM as per our knowledge from the literature and none of those studies worked to identify the significant performance criteria for MSWM technology selection and the cause & effect group criteria as well. In addition to this, the causal interactions among the criteria have not yet been investigated. So, an in-depth understanding of the causal relationships among the performance criteria can provide valuable insight for decision-makers when choosing MSWM technology for Dhaka, Bangladesh.

In summary, the notable contributions of the study are as follows:

• A comprehensive list of significant performance criteria for MSWM technology selection is suggested by integrating various criteria from earlier studies and recommendations of the experts.
• The framework to evaluate the performance criteria for MSWM technology selection is established using a multi-criteria decision-making method.
• Application of the Fuzzy DEMATEL method reveals the interrelationships among the identified criteria and finally, the causal interactions are determined as well.

### 2.6 Rationale behind the study

It is noteworthy to mention that the literature on the implementation of MCDM techniques in the management of municipal solid waste in Bangladesh is very limited as per the background study and Table 3, which highlights the requirements for future scientific research and analysis in this area. That is why, to provide comprehensive insights into sustainable and robust solid waste management practices, the contributions of this study will be significant.

## 3 Methodology

To carry out an effective decision-making process that results in appropriate, dependable, and robust MSW treatment and disposal options, the research starts with a clear problem statement. The present study employs the combination of two different MCDM techniques and consists of two stages; (i) Fuzzy Delphi method (FDM) and (ii) Fuzzy DEMATEL. The fuzzy Delphi method is applied in the first stage to establish the significant performance criteria to select the appropriate MSWM technology and in the second stage, Fuzzy DEMATEL is utilized to identify the cause & effect group criteria and to evaluate the underlying correlations among the identified criteria.

**Fig. 1:**
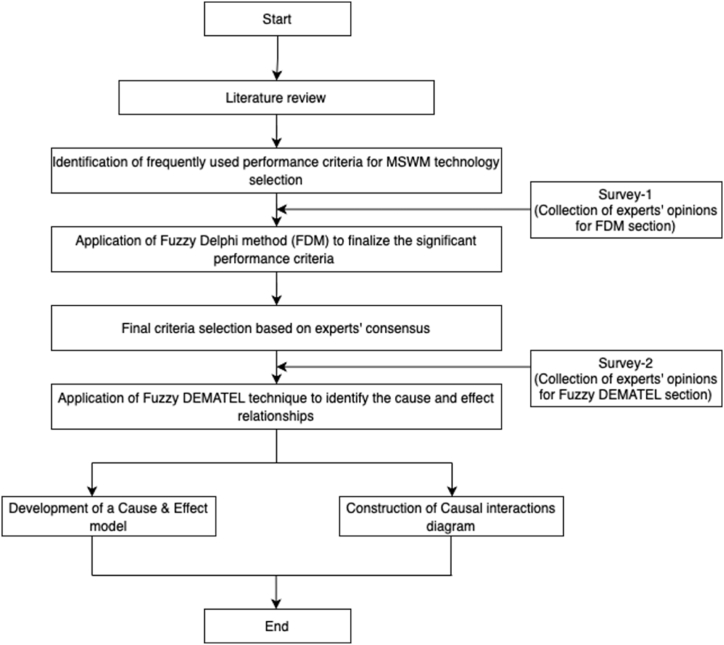
Research Framework used in this study.

Analyzing the current practices of municipal solid waste management in Bangladesh and developing countries ([1,11,12,23,52]), this research considered six alternative solid waste management (treatment and disposal) technologies: (i) Landfilling (LAN), (ii) Composting (COM), (iii) Incineration (INC), (iv) Bio-methanation (BIO), (v) Gasification-pyrolysis (GAS), (vi) Refuse-derived fuel (RDF) combustion. The framework of this research is shown in Fig. 1.

### 3.1 Fuzzy Delphi method (FDM)

#### 3.1.1 Study design

Ishikawa et al. [53] developed the FDM after deriving it from fuzzy set theory and the conventional Delphi method. The implementation of FDM within the framework of group decision-making can effectively remove the ambiguity that typically arises from a shared understanding of expert perspectives. The objective is to ascertain a thorough, functional, non-redundant, and concise compilation of criteria for decision-making that will effectively summarize the diverse range of goals being considered [2].

The present study employed FDM for screening the criteria for the MSWM technology selection process. For addressing the group decision-making issues, triangular fuzzy numbers and functions were employed. In this study, the FDM is employed in three stages: the first stage involves gathering qualitative data from experts through a survey questionnaire; the second stage utilizes the conversion of the qualitative judgments to the Fuzzy numbers and the TFN respectively. Finally, in the third stage, the FDM equations are used and the findings are analyzed.

#### 3.1.2 Data collection and validation

Through the use of reliable sources for data collection, the validity of the current study was ensured. From the literature review of the recent studies, frequently used aspects and criteria for solid waste management of various countries are enlisted. This has to be screened out from the perspective of Bangladesh which is presented in Table 2. The following phase involved consulting expert groups to assess the set of critical criteria for MSWM of Dhaka city. The evaluator groups, consisting of professionals from the academic, research, and experts (from municipal authorities and specialists in the relevant field of consultation) who specialize in the area of solid waste management were approached for their expert opinions to screen the relevant critical criteria from the perspective of Bangladesh. The experts aided in establishing the alternatives and determining the significance of the criteria.

A preliminary survey questionnaire was developed with the aid of three academic experts. Based on the feedback of the group, the questionnaire underwent a process of pre-testing, revision, and validation. The performance criteria were established after the objectives were finalized, and the Fuzzy Delphi method (FDM) was applied to identify the key performance criteria from Bangladesh's perspective. Each decision-maker received the finalized questionnaire where they were asked to rate the importance of each performance criterion. For the purpose of recording, gathering opinions, and arriving at group decisions, interviews were conducted with ten (10) experts from academic and research areas, municipal officials, and waste consultancy firms. Table 4 presents the linguistic scale used for the evaluation and the profile of the involved experts in the FDM section is presented in Tables S–1 (supplementary table**)**. The computing formula for this section is illustrated in Figure. S-2 and Figure. S-3 (supplementary figure**)**.

**Table 4:** Linguistic scale for Fuzzy Delphi analysis (Adapted from Heo et al. [54]).

Linguistic terms	Fuzzy Numbers	Corresponding TFN
Extremely Unimportant	$\tilde{1}$	(0.00,0.00,0.10)
Very Unimportant	$\tilde{2}$	(0.00,0.10,0.30)
Unimportant	$\tilde{3}$	(0.10,0.30,0.50)
Moderately Important	$\tilde{4}$	(0.30,0.50,0.75)
Important	$\tilde{5}$	(0.50,0.75,0.90)
Very Important	$\tilde{6}$	(0.75,0.90,1.00)
Extremely Important	$\tilde{7}$	(0.90,1.00,1.00)

**Fig. 2:**
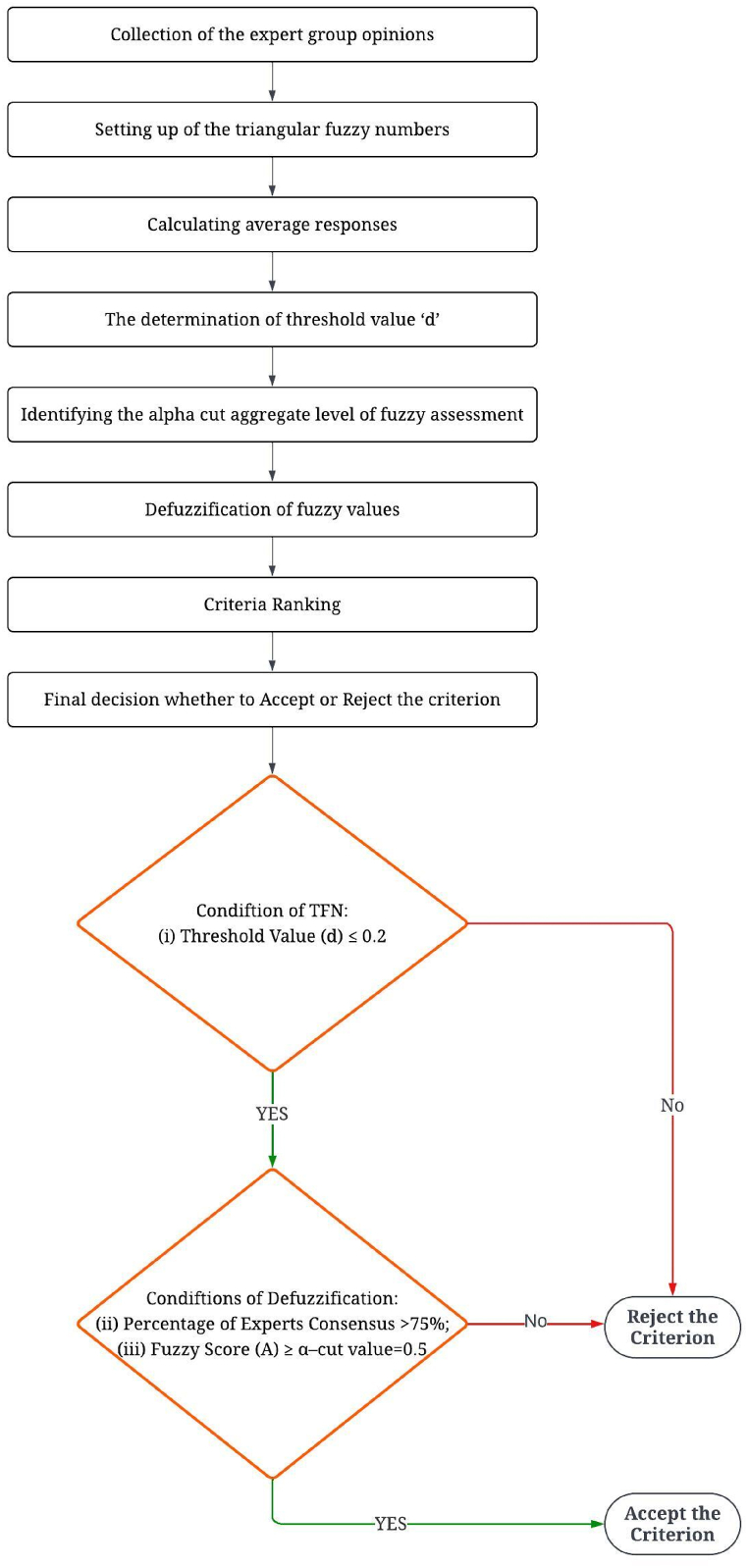
Flowchart of the FDM procedure.

Fig. 2 shows the FDM procedure that was used in this work, which is adapted from Refs. [2,[55], [56], [57]].

Criteria that did not satisfy three conditions: (i) Threshold Value (d) ≤ 0.2; (ii) Percentage of Experts Consensus >75 %; (iii) Fuzzy Score (A) ≥ α – cut value = 0.5, were rejected and thus retaining only 21 criteria from initial 26. The calculation of the FDM procedure is summarized in Tables S–2 (supplementary table**)** and the finalized significant performance criteria are presented in Table 7. Thus, statistical analysis might be used to find more evaluation criteria. The incorporation of the fuzzy theory in the FDM approach was observed to be a more affordable and efficient alternative compared to the traditional Delphi technique.

**Table 5:** Linguistic scale for expert evaluation (Adopted from Priyanka et al. [46]).

Linguistic terms	Influence score	Corresponding TFN
No influence (N)	0	(0.0, 0.1, 0.3)
Very low influence (VL)	1	(0.1, 0.3, 0.5)
Low influence (L)	2	(0.3, 0.5, 0.7)
High influence (H)	3	(0.5, 0.7, 0.9)
Very high influence (VH)	4	(0.7, 0.9, 1.0)

**Table 6:** 

Result summary of fuzzy DEMATEL.	
Criteria with the highest degree of relationship:	T_3_ - Treatment Effectiveness (TE)
Criteria with the highest degree of effect:	**T**_**4**_ - Access to Technology (AT)
Criteria with maximum influence (Giver):	**T**_**1**_ - Technical Reliability (TR)
Criteria with maximum influence (Receiver):	**Ec**_**1**_ - Net Economic Cost (NEC)

**Table 7:** Finalized Performance Criteria with definitions after FDM screening.

Aspects	Criteria	Definitions	Sources
**Technical (T)**	**T**_**1**_ - Technical Reliability (TR)	To function effectively over a set period under specific conditions	[1,2,4,6,8,[11], [12], [13], [14],^16^,^17^,^19^,[26], [27], [28], [29],^31^,^34^,^35^,^37^,^38^]
	**T**_**2**_ - Energy Recovery (ER)	The recoverable potential energy	
	**T**_**3**_-Treatment Effectiveness (TE)	The level of effectiveness of the treatment system	
	**T**_**4**-_ Access to Technology (AT)	The availability of the technology	
	**T**_**5**_-Expert Personnel Requirement (EPR)	Requirement of experts for maintaining the system	
	**T**_**6**-_ Quality and Quantity of Labor (QL)	The skill level of labors and no of employees	
	**T**_**7**-_ Efficiency (E)	The waste volume and weight reduction ratio also known as waste reduction potential	
	**T**_**8**-_ Feasibility (F)	The ability of the disposal system to satisfactorily carry out the desired function	
**Environmental (E)**	**E**_**1**_ - Environmental Feasibility (EF)	The potential of technology to take care of the waste	
	**E**_**2**_ - Air Pollution Control (APC)	Taking into account additional air pollution issues as well as avoiding flue gases that may be produced as a byproduct	
	**E**_**3**_-Water Pollution (WP)	The pollution of surface and groundwater due to the leachate from landfilling and composting facilities	
	**E**_**4**_ -Global Warming (GW)	Global warming rate due to the effect of disposal technology	
	**E**_**5**__–_ Soil Pollution (SP)	The pollution of soil due to the technology	
**Economical (Ec)**	**Ec**_**1**_ – Net Economic Cost/Net Cost per ton of waste (NEC)	All economic costs involved in procuring and implementing the technology	
	**Ec**_**2**_-Initial Investment Cost (IIC)	Set up cost of disposal technologies and their salvage value as well	
	**Ec**_**3**_ - Operational Cost (OC)	Operational expenditures of any specific technology along with their depreciation and maintenance expenses	
	**Ec**_**4**_-Transportation Costs (TC)	Transportation costs related to maintaining the technology	
	**Ec**_**5**_ - Maintenance Cost (MC)	Maintenance costs of the technology installed	
	**Ec**_**6**_-Infrastructure Requirements (IR)	Required infrastructure to develop the technology	
**Social (S)**	**S**_**1**_ - Public Acceptance (PA)	The chosen technology needs to get social acceptance	
	**S**_**2**_ - Awareness (A)	Required public awareness of the system	

### 3.2 Fuzzy DEMATEL

#### 3.2.1 Fuzzy set theory

This study utilizes the combination of fuzzy set theory and the DEMATEL technique to deal with the uncertainty in the collected data for the second stage of this research. To eliminate the uncertainty or to ensure accurate outcomes from human assessments based on fuzzy linguistic variables in the form of fuzzy numbers, a proficient fuzzy aggregation technique that integrates a defuzzification approach is essential.

The fuzzy aggregation method comprises a series of sequential steps [47]:

Step:1 Transferring the linguistic variables of experts' opinions into corresponding triangular fuzzy numbers (TFNs)
Step:2 Normalizing these TFNs
Step:3 Computing the left and right normalized values
Step:4 Acquiring the crisp values
Step:5 Generating the total normalized crisp values
Step:6 Obtaining the direct-relationship matrix by aggregating the normalized crisp values from all experts

#### 3.2.2 DEMATEL method

Decision-making trial and evaluation laboratory (DEMATEL) is a highly functional and advantageous technique for the visualization of complex causal relationships through the utilization of matrices. The matrices or digraphs illustrate a contextual correlation among the elements of the system, wherein a numerical value represents the extent of impact. Hence, it can be inferred that the utilization of the DEMATEL technique has the capability to transform the interdependence among the various cause-and-effect criteria into a clear and organized structural framework of the system [58].

A survey questionnaire was prepared with the help of three academic experts and used to gather opinions from experts for the DEMATEL technique. A group of eleven (11) experts was presented with the input data to analyze the relationships among the identified significant performance criteria and to determine the influential criteria by scoring five linguistic variables: No influence (0), Very low influence (1), Low influence (2), High influence (3), and Very high influence (4). To conduct further analysis, the mean is calculated using reliable data from eleven (11) experts. The data provided requires a higher degree of accuracy for computation with DEMATEL formulas as it is presented in the qualitative form. Therefore, the Fuzzy DEMATEL method is applied which transforms qualitative judgments into crisp numbers through the utilization of triangular fuzzy numbers and then the transformed numbers are used for further calculations of the Fuzzy DEMATEL approach [11,46,59]. After conducting the fuzzy aggregation method as described in Section 3.2.1, the DEMATEL technique is utilized. The DEMATEL methodology for this study is adapted from Refs. [60,61]. The evaluation scale used for this section is represented in Table 5 and the expert teams’ information is presented in Tables S–3 (supplementary table**)** respectively.

The DEMATEL method includes the following steps [60]:

Step:1 Constructing the direct-relationship matrix
Step:2 Normalizing the direct relation matrix
Step:3 Attaining the total relation matrix
Step:4 Calculation of the row and column sums from the total relation matrix
Step:5 Calculation of the overall significance status and net effect values of the criteria
Step:6 Drawing the diagram of the significant importance/effect of DEMATEL and mapping only relationships over a threshold value

## 4 Analysis and results

This study identified twenty-one (21) key performance criteria for selecting the appropriate MSWM technology for Dhaka, Bangladesh through a screening procedure by utilizing FDM. Then, the Fuzzy DEMATEL method was employed to understand the causal relationships among the finalized criteria. Both FDM and the Fuzzy DEMATEL technique were incorporated based on experts’ opinions and judgments.

According to the FDM results shown in Tables S–2 (supplementary table**)**, even though all initial criteria satisfy the condition of receiving a threshold value less than 0.2, only 21 of them obtained the experts’ consensus percentage greater than 75 % and fuzzy score value (A) greater than α-cut value of 0.5, leading to the conclusion that only 21 criteria have attained expert consensus as "Accepted”. Then, the criteria were ranked based on the fuzzy Score value (A), which shows the priority or relevance level of the 26 criteria. The finalized performance criteria are presented in Table 7.

Table 8 represents the findings of the Fuzzy DEMATEL section where the cause-and-effect criteria were classified. After executing the defuzzification procedure and converting the fuzzy numbers into crisp numbers following the guidelines of fuzzy set theory, the residual computations of the Fuzzy DEMATEL segment are provided in Tables S–4 to **S-10 (**supplementary tables**)**. After the computation of the Total Relation matrix (T) as shown in Tables S–9 (supplementary table**),** the vectors *D* and *R*, separately designated to represent the summation of rows and columns are calculated. The summation of rows *(D)* serves as a measure of the impact exerted by specific criteria on other criteria. A higher value for the Row sum *(D)* corresponds to the higher influence and similarly lower the sum indicates low influence, requiring low concentration or attention. Then, the vector denoted as "Prominence”, namely *(D + R)* on the horizontal axis, depicts the extent of significance of the criterion which means a higher value of (*D* + *R*) corresponds to a stronger correlation with other criteria or the highest degree of relationship. Similarly, the vertical axis, denoted as *(D - R)*, is termed as "Relation”, which has the potential to classify criteria into a cause group. The value of *(D - R)* being positive suggests that the criterion belongs to the cause group, whereas a negative value indicates its inclusion in the effect group. The higher value of *(D − R)* possesses the criteria with the highest degree of effect. Therefore, the causal diagram can be obtained through the dataset mapping of *(D + R, D − R)* which facilitates the insight for decision-making. Based on the values of *(D − R)*, the cause group and effect group criteria are determined where the cause group contains fourteen (14) criteria and the effect group contains the remaining seven (7) criteria.

**Table 8:** The prominence and relation axis for the cause-and-effect group.

Criteria	D	R	D + R	D-R	Relationship	Rank
**T**_**1**_	1.3182	0.7337	2.0519	0.5845	Cause	5
**T**_**2**_	1.3694	1.3653	1.3653	0.0042	Cause	14
**T**_**3**_	1.8644	2.3721	2.3721	−0.5077	Effect	16
**T**_**4**_	2.3180	0.2479	0.2479	2.0702	Cause	1
**T**_**5**_	1.0851	1.9779	1.9779	−0.8927	Effect	19
**T**_**6**_	1.4549	1.4038	1.4038	0.0510	Cause	12
**T**_**7**_	1.3896	1.2415	1.2415	0.1481	Cause	9
**T**_**8**_	1.4847	0.7359	0.7359	0.7488	Cause	2
**E**_**1**_	1.3112	0.7200	0.7200	0.5912	Cause	4
**E**_**2**_	0.9343	1.5539	1.5539	−0.6196	Effect	17
**E**_**3**_	1.2875	1.0950	1.0950	0.1924	Cause	8
**E**_**4**_	1.3737	1.3214	1.3214	0.0523	Cause	11
**E**_**5**_	1.3571	1.0778	1.0778	0.2794	Cause	7
**Ec**_**1**_	0.2454	2.1228	2.1228	−1.8774	Effect	21
**Ec**_**2**_	1.4833	1.0396	1.0396	0.4436	Cause	6
**Ec**_**3**_	0.7518	1.7497	1.7497	−0.9979	Effect	20
**Ec**_**4**_	0.6292	0.6212	0.6212	0.0080	Cause	13
**Ec**_**5**_	0.7349	1.3695	1.3695	−0.6346	Effect	18
**Ec**_**6**_	2.3004	1.6503	1.6503	0.6501	Cause	3
**S**_**1**_	0.8819	1.2928	1.2928	−0.4110	Effect	15
**S**_**2**_	1.1868	1.0698	1.0698	0.1170	Cause	10
**Max**	2.3180	2.3721	2.3721	2.0702		
**Min**	0.2454	0.2479	0.2479	−1.8774		

The current investigation determined the criteria that have the most impact on the system by considering each one's position in the whole system which considerably increases the efficiency of the system. From the Row sum *(D),* it can be said that **T**_**3**_-Treatment Effectiveness (TE), **Ec**_**6**_-Infrastructure requirements (IR), and **T**_**4**-_ Access to Technology (AT) are those criteria which have been found to exert the greatest influence on the other criteria. Additionally, it is important to mention that the criteria exhibiting the highest column sum *(R)*, namely, **T**_**3**_-Treatment Effectiveness (TE), **Ec**_**1**_ – Net Economic Cost/Net Cost per ton of wastes (NEC), and **T**_**5**_-Expert Personnel requirement (EPR) receive the highest effect.

It was revealed that the criteria, **T**_**3**_**-** Treatment Effectiveness (TE) exhibited the highest *(D + R)* value of 2.3721 in the group, which indicates the strongest degree of correlation with other criteria and a significant impact on the MSWM technology selection process. Furthermore, the criteria with the greatest degree of effect is **T4-** Access to Technology (AT), as indicated by the highest *(D - R)* value of 2.0702.

Cause group criteria should receive higher attention because they have an effect on the entire system and can influence the overall aim. For example, criteria with the top three higher values of *(D − R)*, **T**_**4**-_ Access to Technology (AT), **T**_**8**-_ Feasibility (F), and the **Ec**_**6**_-Infrastructure requirements (IR), require more attention. These are ranked in the top 3 positions in the Cause group. The summarized results of the Fuzzy DEMATEL section are presented in Table 6.

**Fig. 3:**
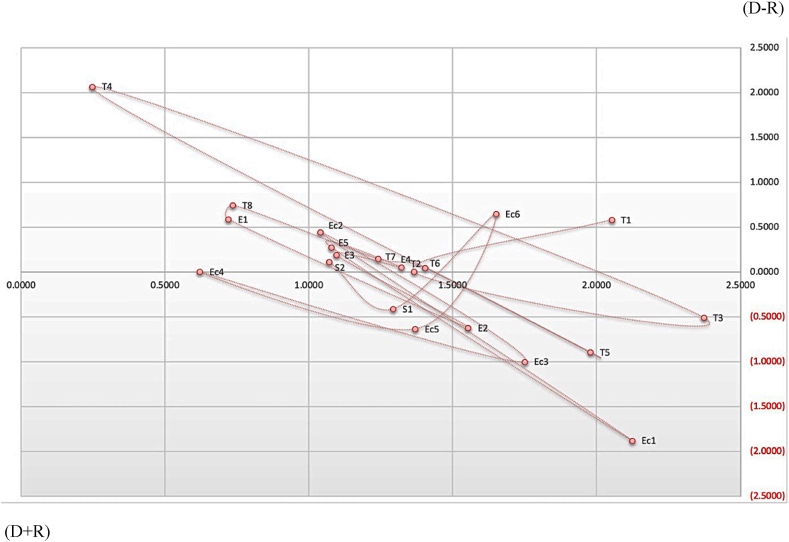
Cause & Effect model.

**Fig. 4:**
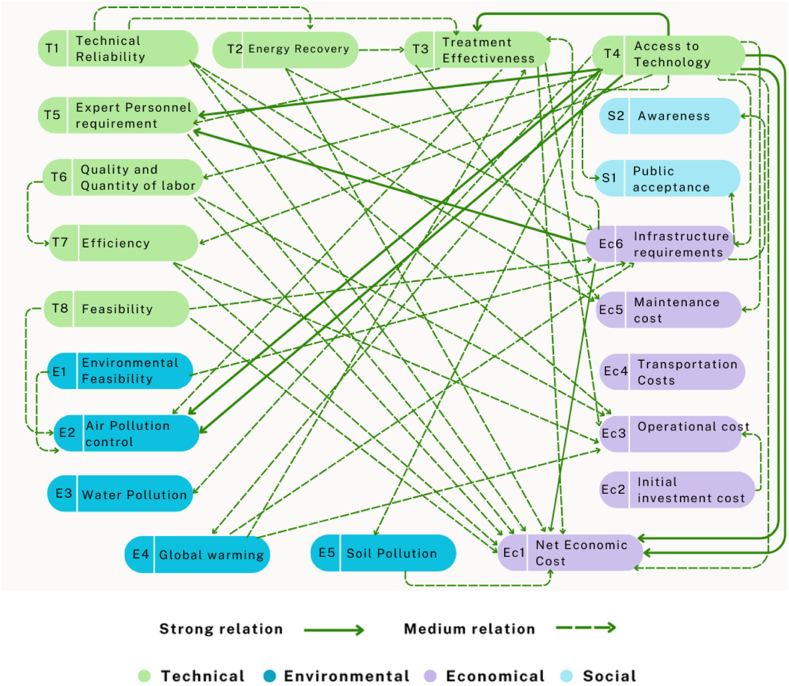
Causal interactions diagram.

A Cause & Effect model is developed and illustrated in Fig. 3 through the process of mapping the dataset of the *(D* + *R, D-R)*, whereby the values of *(D* + *R)* and *(D-R)* are plotted in the X and Y axis respectively. Furthermore, the extent to which the cause criteria influence the effect criteria is also identified using the inner dependence matrix developed in Tables S–10. Based on these findings and the range of the value shown in Tables S–11 (supplementary table**)**, a causal interactions diagram is illustrated in Fig. 4. To enhance the visual representation of the diagram, solely the ‘Strong’ and ‘Medium’ relationships among the criteria have been illustrated. The causal interactions diagram demonstrates the level of influence that each criterion has on the other [62].

## 5 Discussions of the key findings

### 5.1 Cause group

The cause elements indicating the criteria that have more influential characteristics are presented in Fig. 3 and the data of (*D - R*) in Table 8. Depending on its (*D - R*) values, these criteria are ranked as follows: (T_4_>T_8_>Ec_6_>E_1_>T_1_>Ec_2_>E_5_>E_3_>T_7_ >S_2_>E_4_>T_6_>Ec_4_>T_2_). The selection of an MSWM technology requires greater consideration of these criteria. The influence increases as the *(D - R)* value increases. **T**_**4**-_ Access to Technology (AT) is ranked first since it has the highest value of *(D - R)*, and all other criteria are influenced by it. This indicates that the process of choosing an MSWM technology is influenced by the availability of the specific technology. Similarly, based on the sequence of their influential behaviour, other cause elements existing in the cause group also require higher consideration.

### 5.2 Effect group

Fig. 3 and the data of *(D - R)* in Table 8 demonstrate the effect elements indicating the criteria with less influential characteristics. Based on its (*D - R*) values, these criteria are ranked as follows: (S_1_>T_3_>E_2_>Ec_5_>T_5_>Ec_3_> Ec_1_). Containing the lower (*D - R*) value means the lower influence of these criteria and they require less attention than the cause group criteria because of their comparatively less impactful behaviour. Many effect elements will be eliminated as a result of reducing the cause elements, which will benefit the effect group without requiring special consideration. For example, **Ec**_**1**_– Net Economic Cost/Net Cost per ton of wastes (NEC), the lowermost criterion in the effect group, represents all economic costs involved in procuring and implementing the technology, can be resolved by dealing with the causal elements, **Ec**_**2**_-Initial investment cost (IIC), **Ec**_**4**_-Transportation Costs (TC) and **Ec**_**6**_-Infrastructure requirements (IR). The net economic cost for any specific technology will naturally reduce if the initial investment, transportation, and infrastructure expenses are minimal. This will reduce the special attention needed for the criterion, **Ec**_**1**_–Net Economic Cost as a result. Similarly, when addressing cause elements with respect to their influential rank order, it may be possible to eliminate all other elements in the effect group.

### 5.3 Relationships among the criteria

The (*D + R*) value is shown in Table 8, which represents the high relation value of the criteria. It has a significant influence over other criteria and is influenced by others. Based on the value of (*D + R*), the criteria are subsequently ranked in a hierarchical order as stated below:

(T_3_>Ec_1_>T_1_>T_5_>Ec_3_>Ec_6_>E_2_>T_6_>Ec_5_>T_2_>E_4_>S_1_>T_7_>E_3_>E_5_>S_2_>Ec_2_>T_8_>E_1_>Ec_4_>Ec_6_). Higher the (*D + R*) value, the higher the degree of relationship. Here, the criteria, **T**_**3**_-Treatment Effectiveness (TE), **Ec**_**1**_ – Net Economic Cost/Net Cost per ton of wastes (NEC), and **T**_**1**_ - Technical Reliability (TR), are listed in the top three places and reflect their stronger relationships with other criteria as well as their greater influence.

### 5.4 Causal interactions

Based on the developed Tables S–10 and Tables S–11 (supplementary table**)**, the causal interaction diagram is presented in Fig. 4. This highlights two distinct types of relationships, namely strong and medium between the cause group and effect group elements. The diagram effectively demonstrates the extent to which the cause elements affect the effect group elements. It reveals that the first cause criteria, **T**_**1**_ - Technical Reliability (TR) has a medium effect on 4 criteria (**T**_**3**_ **-** Treatment Effectiveness, **Ec**_**1**_**-** Net Economic Cost, **Ec**_**3**_ - Operational cost, and **Ec**_**5**_ - Maintenance cost (MC)) from effect group elements and has a weak or low effect on another 5 criteria (**T**_**2**_ - Energy Recovery (ER), **T**_**8**-_ Feasibility (F), **E**_**1**_ - Environmental Feasibility (EF), **E**_**2**_ - Air Pollution control (APC), **E**_**4**_ -Global warming (GW)). However, it does not affect the other eleven remaining criteria. This indicates that the treatment effectiveness, net economic cost, operational cost, and maintenance cost for that system will be moderately affected if any specific technology is not technically reliable. Similarly, the last cause criteria, **S**_**2**_ - Awareness (A) receives the medium effect from **Ec**_**6**_-Infrastructure requirements (IR). The criteria, **T**_**4**-_ Access to Technology (AT) influences the maximum elements of the list which implies that the availability of the specific technology has huge influences on the performance of the entire system and overall technology selection process. However, **Ec**_**1**_ – Net Economic Cost/Net Cost per ton of wastes (NEC) receives the strongest impact from **Ec**_**6**_-Infrastructure requirements (IR) as well as the moderate influence from many other criteria. This indicates the net economic cost of the system fluctuates depending on the infrastructure needs and related costs and can be eliminated by dealing with the causal elements.

### 5.5 Comparative analysis

In order to illustrate the comparative analysis, the proposed method and outcomes of this paper are compared with similar studies from different countries. As summarized in Table 3, some of the recent studies from different countries with similar socio-economic and environmental conditions applied MCDM approaches like Delphi, AHP, BWM, PROMOTHEE, TOPSIS, VIKOR, CRITIC-MULTIMOORA, DEMATEL, CODAS and others. But the majority of these studies including ([12,34,38,42,48,63]) did not consider uncertainty in the opinions and judgements of the experts. In comparison with the existing studies, this research considers the fuzzy-based methods in both the first and second stages to eliminate the uncertainty from the data. The proposed approach of this study integrates the Fuzzy Delphi and Fuzzy DEMATEL-based methodology for the data validation and more accurate analysis which differentiates the current study from the existing others.

Numerous studies considered very few aspects and criteria for the technology alternative evaluation, landfill site selection and other waste management initiatives. Such as a recent study [63] considers only five (5) criteria for selecting solid waste disposal site and another two recent studies ([35,37]) considers nine (9) sub-criteria for selecting plastic waste treatment technology and waste collection truck respectively. Thus, previous studies like ([2,11]) evaluate technology alternatives and landfill site selection against twelve (12) relevant criteria. Conversely, our study includes four different aspects (Technological, Environmental, Economical and Social) and analyzes interdependencies among a set of twenty-one (21) significant criteria for creating a pathway in selecting appropriate technology alternatives.

Prior studies focused on several waste management initiatives including disposal site selection, landfill site selection, evaluation and comparison of disposal and treatment technology alternatives. Therefore, the present study mainly focuses on analyzing the interdependencies among the performance criteria which creates a decision-making framework for choosing the best MSWM technology alternative. In comparison with the outcomes from the existing studies ([38,[63], [64], [65], [66]]) regarding the evaluation and selection of the best alternative from a set of options, our study draws a structured route to select the appropriate MSWM technology alternative based on our analysis and findings. Several criteria, including technical reliability, feasibility, water pollution, and soil pollution, have been identified by Qinghua et al. [37] and our analysis as the causal factors, along with feasibility and technical reliability being found as significant. In terms of addressing air pollution, our study has determined the air pollution control is an effect factor while Qinghua et al. reported the air pollution as a causal factor. Similarly, Manoj et al. [11] utilized the Fuzzy-AHP method and identified the net economic cost of the system, environmental feasibility, energy recovery and technical reliability as the significant criteria for evaluating technology alternatives whereas our analysis found environmental feasibility as the least significant and net economic cost as the criteria which receives the maximum influences. In the present research, energy recovery and technical reliability have been found to be significant criteria. Thus, in this particular domain, the present research offers unique findings and an additional illustration of the causal interactions to know the extent of the influence of any specific criteria which needs to be considered to generate the best decision.

## 6 Conclusions, implications, and limitations

### 6.1 Conclusions

The significance of employing suitable management technology practices for the effective management of MSW is readily apparent for any nation, particularly for developing countries such as Bangladesh, which confront various challenges in the domain of MSW management. However, developing countries (i.e., Bangladesh) do struggle with the selection of effective and appropriate MSWM technology. This paper has therefore attempted to identify, evaluate, and prioritize the MSWM technology selection criteria as well as investigate the relationship between these criteria and the extent to which they influence or are influenced by one another, using an integrated Fuzzy-Delphi-Fuzzy-DEMATEL methodology. The study identifies and analyzes a set of 21 significant performance criteria, which need to be considered in the selection process. To get more logical and accurate findings, the established methodology efficiently accounts for the ambiguous, subjective, and linguistic data from expert viewpoints. Our proposed methodology has the potential to accurately quantify this specific data. It includes an advantageous method for weighting the criteria according to their level of significance, influence and interrelation with other criteria, which directs the decision-makers to find the most appropriate solution.

The FDM extensively gathers data related to various aspects and effectively addresses the lack of clarity and ambiguity that exists in the assessments carried out by experts, with the goal of establishing the critical criteria for evaluation. The Fuzzy DEMATEL methodology ascertains the ranking of the evaluation criteria, prioritizes them and determines the interdependent relationship among the identified criteria. The Cause & Effect model depicted by DEMATEL enables decision-makers to identify the dimension and criterion that acts as the dispatcher, exerting influence on other dimensions and criteria within a given system. Furthermore, the causal interactions diagram which was generated based on the findings of the Fuzzy DEMATEL section, presents the degree of influence of the causal elements and a better understanding of which criterion depends on others in the system, that gives insight for sound decision-making.

From the result of Fuzzy DEMATEL, it is clear that Treatment Effectiveness (TE) or the effectiveness of any specific treatment facility have the highest degree of relationship with other criteria in the system. Then, Net Economic Cost (NEC) or the total associated costs needed for any treatment facility, receives maximum influence from other technology selection criteria. This implies that the net economic cost for the system will be reduced automatically if the influencing causal elements are handled properly. Access to Technology (AT) comes out to be a dimension having the highest degree of effect. This denotes that the presence of a particular technology has a greater impact on the overall systems’ performance and the process of technology selection.

The main findings of this research offer significant and valuable observations into the performance criteria that are critical and crucial for the municipal solid waste management technology selection process, and the understanding of how these criteria are interrelated. Interrelationships among the criteria provide a better understanding of each criterion's role in the MSWM technology selection process. Moreover, the combination of Fuzzy set theory with DEMATEL results in the elimination of the fuzziness and imprecision in the expert's judgments indicating that it is a beneficial technique for improving problem comprehension and assisting decision-makers in reliable decision-making. The outcomes of the investigation will help the decision-makers to get a comprehensive understanding of the interrelationships among the criteria. This understanding is imperative for effective management of MSW as well as for selecting the best alternative based on their own facilities. Thus, the proposed method presented in this paper can be applied to select the appropriate technology for managing MSW as it adopts a holistic approach to address the real-world problem. Furthermore, it has been ascertained that the recommended analytical procedure is proficient in addressing the issue of technology selection involving multi-faceted factors as it combines multiple methodologies and advanced techniques to tackle the complexities of MSWM comprehensively.

### 6.2 Research implications

The theoretical implications of this research are numerous. For instance, it has the ability to make a significant literary contribution to the process of selecting MSWM technology by identifying the key performance criteria, ranking the criteria, and outlining the relationships between them within the framework of emerging economies. This study can serve as a fundamental base for the researchers to investigate the implementation of MSWM technology in emerging economies. However, this study has the potential to make a valuable addition to the current understanding of MSWM technology selection in Dhaka, the capital city of Bangladesh, and could prove beneficial for other countries facing similar technological, environmental, and socio-economic circumstances.

The research holds critical significance and several practical implications for managers and decision-makers involved with solid waste management in Bangladesh and other developing countries. Firstly, it can reveal a systematic plan to select an appropriate MSWM technology. Secondly, the study can assist the decision-makers in evaluating the MSWM technology alternatives, which can improve productivity, efficiency, and the selection of sustainable management technology.

### 6.3 Limitations of the study and future research scopes

The findings of this study are only applicable to the cities and developing countries with comparable technological, environmental, and socio-economic conditions to Dhaka, Bangladesh because our focus has examined those specific criteria for this area only. Future studies may comprise all other areas from Bangladesh and will identify and investigate the relationships among the applicable criteria for those areas. This study has included a limited set of performance criteria from previous studies and relevant experts which could potentially lead to suboptimal decisions. Hence, we recommend including more relevant and new performance criteria for the MSWM technology selection process and analysing them in future studies. Only six MSW management technology alternatives (Landfilling, Composting, Incineration, Bio-methanation, Gasification-pyrolysis, and Refuse-derived fuel combustion) are considered for the current investigation. Therefore, it is recommended to explore additional advanced treatment technologies in future investigations. Additionally, forthcoming research can evaluate the benefits of each technology alternative in light of the current study's findings and determine which technologies are the most advantageous. In dynamic environments where real-time data is crucial, the research framework might not be the best fit. MSWM technologies and their performance may change over time, and the framework may not adapt quickly to these changes. Our current sample size is relatively small and future studies may benefit from utilizing larger sample sizes as data limitations can affect the reliability of the results. Even though the fuzzy set theory is used to manage uncertainty in decision-making, the choice of MSWM technology, in the end, may still be subjective due to differing expert opinions and the theory's potential to manage uncertainty rather than totally eradicate it. That is why, a comparative study applying other advanced MCDM techniques can be done to get comparative insights for more accurate outcomes.

## Funding acknowledgement

This research did not receive any specific grant from funding agencies in the public, commercial, or not-for-profit sectors.

## Data availability statement

Data will be made available on reasonable request.

## CRediT authorship contribution statement

**Mehedi Hasan Shanta:** Writing – original draft, Visualization, Validation, Software, Methodology, Investigation, Formal analysis, Data curation, Conceptualization. **Imtiaz Ahmed Choudhury:** Writing – review & editing, Supervision. **Sheak Salman:** Writing – review & editing, Resources.

## Declaration of competing interest

The authors declare that they have no known competing financial interests or personal relationships that could have appeared to influence the work reported in this paper.
