# Supplementary material for: Municipal solid waste management: Identification and analysis of technology selection criteria using Fuzzy Delphi and Fuzzy DEMATEL technique
Source: Heliyon. 2023 Dec 5;10(1):e23236. doi: 10.1016/j.heliyon.2023.e23236 (PMC10754890; doi:10.1016/j.heliyon.2023.e23236)
Supplement: Multimedia component 1 [file mmc1.docx]

**Ahsanullah University of Science and Technology, Dhaka, Bangladesh**

**Department of Mechanical and Production Engineering**

**Survey Questionnaire-1**

Respected professional,

In this Survey, we are collecting Expert's opinions to figure out the significant performance criteria in order to select municipal solid waste management technology for Dhaka, Bangladesh. The title of the research is **‘’Municipal solid waste management: Identification and analysis of technology selection criteria using Fuzzy Delphi and Fuzzy DEMATEL technique''**. The Experts involved in this research will be Academicians and Municipal officials. This research is purely academic research. All the contents of the questionnaire are strictly confidential and are only for this research discussion.

From the literature review of the recent studies done in different countries specially the developing countries like Bangladesh, we enlisted frequently used Aspects and Performance Criteria for municipal solid waste management which need to be screened from the perspective of Bangladesh.

To improve the existing waste management system of Bangladesh and for the sake of our research, we will be delighted to have your co-operation with us if you provide your valuable opinions and comments. The survey will take about 20 minutes to complete and we sincerely invite you to give us your insights as a reference for this research. Thank you for your patience in helping to fill out the answers and for your generous advice as well. Your support will be the key to the success of this research. Please fill in with confidence based on your experience and actual condition. **Table 1** below presents the measurement scale you will have to consider to identify the level of the importance of any specific criterion and **Table 2** presents the definitions of the criteria presented for your evaluation. At the end of the evaluation, you will find a blank for your suggestions and recommendations for

Kindly fill up your necessary information mentioned below:

1. Name:
2. Designation:
3. Company name:
4. Years of experience:

**Table 1** Measurement Scale for evaluation

| **Evaluation score** | **The extent of the impact** |
| --- | --- |
| 1 | Extremely unimportant |
| 2 | Very unimportant |
| 3 | Unimportant |
| 4 | Moderately Important |
| 5 | Important |
| 6 | Very important |
| 7 | Extremely important |

**Table 3** Definitions of the Criteria

| **Aspects** | **Criteria** | **Definitions** |
| --- | --- | --- |
| **Technical (T)** | Technical Reliability (TR) | To function effectively over a set period of time under specific conditions |
|  | Energy Recovery (ER) | The recoverable potential energy |
|  | Treatment Effectiveness (TE) | The level of effectiveness of the treatment system |
|  | Access to Technology (AT) | The availability of the technology |
|  | Power generation rate (PGR) | Rate of power achieved from the system |
|  | Efficiency (E) | The waste volume and weight reduction ratio also known as waste reduction potential |
|  | Feasibility (F) | Ability of the disposal system to satisfactorily carry out the desired function |
| **Environmental (E)** | Environmental Feasibility (EF) | The potential of technology to take care of the waste |
|  | Air Pollution control (APC) | Taking into account additional air pollution issues as well as avoiding flue gases that may be produced as a byproduct |
|  | Emission Control levels (ECL) | Avoiding the release of GHGs, furans, heavy metals etc. |
|  | Water Pollution (WP) | The pollution of surface and groundwater due to the leachate from landfilling and composting facilities |
|  | Global warming (GW) | Global warming rate due to the effect of disposal technology |
|  | Soil Pollution (SP) | The pollution of soil due to the technology |
|  | SOX and NOX emissions (SNE) | The emission rate of SOX and NOX gases due to the technology |
| **Economical (Ec)** | Net Economic Cost/ Net Cost per ton of wastes (NEC) | All economic costs involved in procuring and implementing of the technology |
|  | Operational cost (OC) | Operational expenditures of any specific technology along with their depreciation and maintenance expenses |
|  | Revenues (R) | Revenue from the technology installed |
|  | Transportation Costs (TC) | Transportation costs related to maintaining the technology |
|  | Maintenance cost (MC) | Maintenance costs of the technology installed |
| **Social (S)** | Public acceptance (PA) | The chosen technology needs to get social acceptance |
|  | Political support (PS) | Political support of the technology |
|  | Awareness (A) | Public Awareness required for the system |

**Table 3** Mark (**
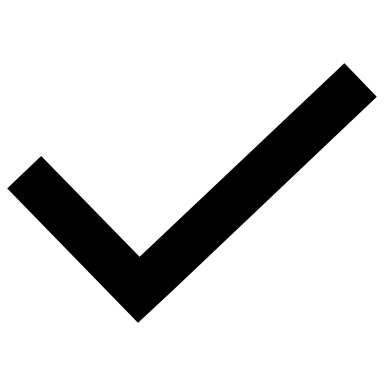
**) the level of importance for below mentioned criteria as per the measurement scale mentioned in Table 1.

| **Aspects** | **Criteria** | **Evaluation scale** | | | | | | |  |
| --- | --- | --- | --- | --- | --- | --- | --- | --- | --- |
|  |  | Extremely unimportant | Very unimportant | Unimportant | Moderately Important | Important | Very important | Extremely important |  |
| **Technical (T)** | Technical Reliability (TR) |  |  |  |  |  |  |  |  |
|  | Energy Recovery (ER) |  |  |  |  |  |  |  |  |
|  | Treatment Effectiveness (TE) |  |  |  |  |  |  |  |  |
|  | Access to Technology (AT) |  |  |  |  |  |  |  |  |
|  | Power generation rate (PGR) |  |  |  |  |  |  |  |  |
|  | Efficiency (E) |  |  |  |  |  |  |  |  |
|  | Feasibility (F) |  |  |  |  |  |  |  |  |
| **Environmental (E)** | Environmental Feasibility (EF) |  |  |  |  |  |  |  |  |
|  | Air Pollution control (APC) |  |  |  |  |  |  |  |  |
|  | Emission Control levels (ECL) |  |  |  |  |  |  |  |  |
|  | Water Pollution (WP) |  |  |  |  |  |  |  |  |
|  | Global warming (GW) |  |  |  |  |  |  |  |  |
|  | Soil Pollution (SP) |  |  |  |  |  |  |  |  |
|  | SOX and NOX emissions (SNE) |  |  |  |  |  |  |  |  |
| **Economical (Ec)** | Net Economic Cost/ Net Cost per ton of wastes (NEC) |  |  |  |  |  |  |  |  |
|  | Operational cost (OC) |  |  |  |  |  |  |  |  |
|  | Revenues (R) |  |  |  |  |  |  |  |  |
|  | Transportation Costs (TC) |  |  |  |  |  |  |  |  |
|  | Maintenance cost (MC) |  |  |  |  |  |  |  |  |
| **Social (S)** | Public acceptance (PA) |  |  |  |  |  |  |  |  |
|  | Political support (PS) |  |  |  |  |  |  |  |  |
|  | Awareness (A) |  |  |  |  |  |  |  |  |
| Please suggest if any other relevant and necessary Performance Criteria are missing |  | | | | | | | | |

**_____________________ _______________**

**Signature Date**
