# Supplementary material for: Municipal solid waste management: Identification and analysis of technology selection criteria using Fuzzy Delphi and Fuzzy DEMATEL technique
Source: Heliyon. 2023 Dec 5;10(1):e23236. doi: 10.1016/j.heliyon.2023.e23236 (PMC10754890; doi:10.1016/j.heliyon.2023.e23236)
Supplement: Multimedia component 2 [file mmc2.docx]

**Ahsanullah University of Science and Technology, Dhaka, Bangladesh**

**Department of Mechanical and Production Engineering**

**Survey Questionnaire-2**

Respected professional,

In this Survey, we are collecting Expert's opinions to identify the interrelationships among 21 significant performance criteria in order to select municipal solid waste management technology for Dhaka, Bangladesh. The title of the research is **‘’Municipal solid waste management: Identification and analysis of technology selection criteria using Fuzzy Delphi and Fuzzy DEMATEL technique''**. The Experts involved in this research will be Academicians and Municipal officials. This research is purely academic research. All the contents of the questionnaire are strictly confidential and are only for this research discussion.

To improve the existing waste management system of Bangladesh and for the sake of our research, we will be delighted to have your co-operation with us if you provide your valuable opinions and comments. The survey will take about 30 minutes to complete and we sincerely invite you to give us your insights as a reference for this research. Thank you for your patience in helping to fill out the answers and for your generous advice as well. Your support will be the key to the success of this research. Please fill in with confidence based on your experience and actual condition.

You will have to consider six (6) solid waste management (treatment and disposal) technology alternatives while you are evaluating the interdependency of the criteria or the degree of their mutual influence. The alternatives and their definitions are presented in **Table 1**. Table 2 presents the evaluation scale.

Kindly fill up your necessary information mentioned below:

1. Name:
2. Designation:
3. Company name:
4. Years of experience:

**Table 1** Descriptions of Solid Waste Management Technologies Considered in the study

| **Technology alternatives** | **Description** |
| --- | --- |
| Landfilling (LAN) | The systematic burial of wastes which is the final placement of waste into or onto the land in a controlled way |
| Composting (COM) | Compost is the mixture of ingredients used as plant fertilizer and to improve soil's physical, chemical and biological properties |
| Incineration (INC) | Incineration is a waste treatment process that involves the combustion of substances contained in waste materials |
| Bio-methanation (BIO) | Bio-methanation is a process by which organic material is microbiologically converted under anaerobic conditions to biogas |
| Gasification-pyrolysis (GAS) | Gasification and pyrolysis are thermal processes for converting carbonaceous substances into tar, ash, coke, char, and gas |
| Refuse-derived fuel (RDF) combustion | Production of RDF through the combustion of solid wastes |

**Instruction for filling out the questionnaire:**

The questionnaire of this research is divided into four parts:

1. Evaluation scale and Definitions of Performance Criteria
2. Questionnaire filling instruction
3. Interrelationships table fill up
4. Basic information

When someone asks “How are you feeling today?” We express our feelings in a qualitative perception rather than a quantitative manner. And our qualitative perception is fuzzy means it is not precise also it contains uncertainty. So, to have reliable data we have to collect perceptions in qualitative terms and then we have to address the fuzziness in quantitative terms.

**1. Evaluation scale and Definitions of Performance Criteria**

**Table 2** Evaluation Scale for influence identification

| **Linguistic terms** | **The extent of the influence** | **Explanation** | **Indication** |
| --- | --- | --- | --- |
| No influence | Has no influence | Represents that two evaluation criteria are not related to each other | N |
| Very low influence | Very Low influence | Represents a low correlation between the two evaluation criteria | VL |
| Low influence | Low influence | Represents a poor correlation between the two evaluation criteria | L |
| High influence | High influence | Represents a high degree of correlation between the two evaluation criteria | H |
| Very high influence | Very High influence | Represents a very high degree of correlation between the two evaluation criteria | VH |

**Table 3** Performance criteria with definitions

| **Asp.** | **Criteria** | **Definitions** |
| --- | --- | --- |
| **Technical (T)** | **T_1_** - Technical Reliability (TR) | To function effectively over a set period of time under specific conditions |
|  | **T_2_** - Energy Recovery (ER) | The recoverable potential energy |
|  | **T_3_** _-_ Treatment Effectiveness (TE) | The level of effectiveness of the treatment system |
|  | **T_4_**_-_ Access to Technology (AT) | The availability of the technology |
|  | **T_5_** _-_ Expert Personnel requirement (EPR) | Requirement of experts for maintaining the system |
|  | **T_6_**_-_ Quality and Quantity of labor (QL) | The skill level of labors and no of employees |
|  | **T_7_**_-_ Efficiency (E) | The waste volume and weight reduction ratio also known as waste reduction potential |
|  | **T_8_**_-_ Feasibility (F) | Ability of the disposal system to satisfactorily carry out the desired function |
| **Environmental (E)** | **E_1_** - Environmental Feasibility (EF) | The potential of technology to take care of the waste |
|  | **E_2_** - Air Pollution control (APC) | Taking into account additional air pollution issues as well as avoiding flue gases that may be produced as a byproduct |
|  | **E_3_** _-_ Water Pollution (WP) | The pollution of surface and groundwater due to the leachate from landfilling and composting facilities |
|  | **E_4_** _-_Global warming (GW) | Global warming rate due to the effect of disposal technology |
|  | **E_5_** _–_ Soil Pollution (SP) | The pollution of soil due to the technology |
| **Economical (Ec)** | **Ec_1_** – Net Economic Cost/ Net Cost per ton of wastes (NEC) | All economic costs involved in procuring and implementing of the technology |
|  | **Ec_2_** _-_ Initial investment cost (IIC) | Set up cost of disposal technologies and their salvage value as well |
|  | **Ec_3_** - Operational cost (OC) | Operational expenditures of any specific technology along with their depreciation and maintenance expenses |
|  | **Ec_4_** _-_ Transportation Costs (TC) | Transportation costs related to maintaining the technology |
|  | **Ec_5_** - Maintenance cost (MC) | Maintenance costs of the technology installed |
|  | **Ec_6_**-Infrastructure requirements (IR) | Required infrastructure to develop the technology |
| **Social (S)** | **S_1_** - Public acceptance (PA) | The chosen technology needs to get social acceptance |
|  | **S_2_** - Awareness (A) | Public Awareness required for the system |

**2. Questionnaire filling instruction**

Fill in the example, as shown in the table below:

(1) Based on your expertise, if you believe that A has “No influence” on C, please fill in **‘’N’’** in the box and if A has ‘’High influence’’ on C, please fill in ‘’**H**’’.

(2) Based on your expertise, if you believe that B has a ‘’Low influence’’ and “Very High influence” on B and C respectively, please fill ‘’**L**’’ and **‘’VH’’** in the box

(3) If you believe that C has ‘Very low influence’’ on A and B, then fill ‘’**VL**’’ in the box.

| **Affected by**  **(effects)**  **The main**  **Influence**  **(cause)** | **A** | **B** | **C** |
| --- | --- | --- | --- |
| **A** | The effect of A on A does not need to be filled in | **N** | **H** |
| **B** | **L** | The effect of B on B does not need to be filled in | **VH** |
| **C** | **VL** | **VL** | The effect of C on C does not need to be filled in |

**3. Interrelationships table fill up**

As per instructions stated above and evaluation scale provided in Table 1, fill out the table below.

|  | T1- Technical Reliability  **Main Influence**  **cause**  **Affected Criteria** | T2- Energy Recovery | T3- Treatment Effectiveness (TE) | T4- Access to Technology (AT) | T6- Expert Personnel requirement (EPR) | T7- Quality and Quantity of labor (QL) | T8- Efficiency (E) | T9- Feasibility (F) | E1- Environmental Feasibility (EF) | E2- Air Pollution Control (APC) | E4- Water Pollution (WP) | E5- Global warming (GW) | E6- Soil Pollution (SP) | Ec1- Net Economic Cost (NEC) | Ec2- Initial Investment Cost (IIC) | Ec3- Operational Cost (OC) | Ec5- Transportation Cost (TC) | Ec6- Maintenance Cost (MC) | Ec7- Infrastructure requirements (IR) | S1- Public acceptance (PA) | S3- Awareness (A) |
| --- | --- | --- | --- | --- | --- | --- | --- | --- | --- | --- | --- | --- | --- | --- | --- | --- | --- | --- | --- | --- | --- |
| T1- Technical Reliability |  |  |  |  |  |  |  |  |  |  |  |  |  |  |  |  |  |  |  |  |  |
| T2- Energy Recovery |  |  |  |  |  |  |  |  |  |  |  |  |  |  |  |  |  |  |  |  |  |
| T3- Treatment Effectiveness (TE) |  |  |  |  |  |  |  |  |  |  |  |  |  |  |  |  |  |  |  |  |  |
| T4- Access to Technology (AT) |  |  |  |  |  |  |  |  |  |  |  |  |  |  |  |  |  |  |  |  |  |
| T5- Expert Personnel requirement (EPR) |  |  |  |  |  |  |  |  |  |  |  |  |  |  |  |  |  |  |  |  |  |
| T6- Quality and Quantity of labor (QL) |  |  |  |  |  |  |  |  |  |  |  |  |  |  |  |  |  |  |  |  |  |
| T7- Efficiency (E) |  |  |  |  |  |  |  |  |  |  |  |  |  |  |  |  |  |  |  |  |  |
| T8- Feasibility (F) |  |  |  |  |  |  |  |  |  |  |  |  |  |  |  |  |  |  |  |  |  |
| E1- Environmental Feasibility (EF) |  |  |  |  |  |  |  |  |  |  |  |  |  |  |  |  |  |  |  |  |  |
| E2- Air Pollution Control (APC) |  |  |  |  |  |  |  |  |  |  |  |  |  |  |  |  |  |  |  |  |  |
| E3- Water Pollution (WP) |  |  |  |  |  |  |  |  |  |  |  |  |  |  |  |  |  |  |  |  |  |
| E4- Global warming (GW) |  |  |  |  |  |  |  |  |  |  |  |  |  |  |  |  |  |  |  |  |  |
| E5- Soil Pollution (SP) |  |  |  |  |  |  |  |  |  |  |  |  |  |  |  |  |  |  |  |  |  |

|  | T1- Technical Reliability | T2- Energy Recovery | T3- Treatment Effectiveness (TE) | T4- Access to Technology (AT) | T6- Expert Personnel requirement (EPR) | T7- Quality and Quantity of labor (QL) | T8- Efficiency (E) | T9- Feasibility (F) | E1- Environmental Feasibility (EF) | E2- Air Pollution Control (APC) | E4- Water Pollution (WP) | E5- Global warming (GW) | E6- Soil Pollution (SP) | Ec1- Net Economic Cost (NEC) | Ec2- Initial Investment Cost (IIC) | Ec3- Operational Cost (OC) | Ec5- Transportation Cost (TC) | Ec6- Maintenance Cost (MC) | Ec7- Infrastructure requirements (IR) | S1- Public acceptance (PA) | S3- Awareness (A) |
| --- | --- | --- | --- | --- | --- | --- | --- | --- | --- | --- | --- | --- | --- | --- | --- | --- | --- | --- | --- | --- | --- |
| **Affected Criteria**  **Main Influence**  **cause**  Ec1- Net Economic Cost (NEC) |  |  |  |  |  |  |  |  |  |  |  |  |  |  |  |  |  |  |  |  |  |
| Ec2- Initial Investment Cost (IIC) |  |  |  |  |  |  |  |  |  |  |  |  |  |  |  |  |  |  |  |  |  |
| Ec3- Operational Cost (OC) |  |  |  |  |  |  |  |  |  |  |  |  |  |  |  |  |  |  |  |  |  |
| Ec4- Transportation Cost (TC) |  |  |  |  |  |  |  |  |  |  |  |  |  |  |  |  |  |  |  |  |  |
| Ec5- Maintenance Cost (MC) |  |  |  |  |  |  |  |  |  |  |  |  |  |  |  |  |  |  |  |  |  |
| Ec6- Infrastructure requirements (IR) |  |  |  |  |  |  |  |  |  |  |  |  |  |  |  |  |  |  |  |  |  |
| S1- Public acceptance (PA) |  |  |  |  |  |  |  |  |  |  |  |  |  |  |  |  |  |  |  |  |  |
| S2- Awareness (A) |  |  |  |  |  |  |  |  |  |  |  |  |  |  |  |  |  |  |  |  |  |
