# Supplementary figures and images for: Municipal solid waste management: Identification and analysis of technology selection criteria using Fuzzy Delphi and Fuzzy DEMATEL technique
Source: Heliyon. 2023 Dec 5;10(1):e23236. doi: 10.1016/j.heliyon.2023.e23236 (PMC10754890; doi:10.1016/j.heliyon.2023.e23236)

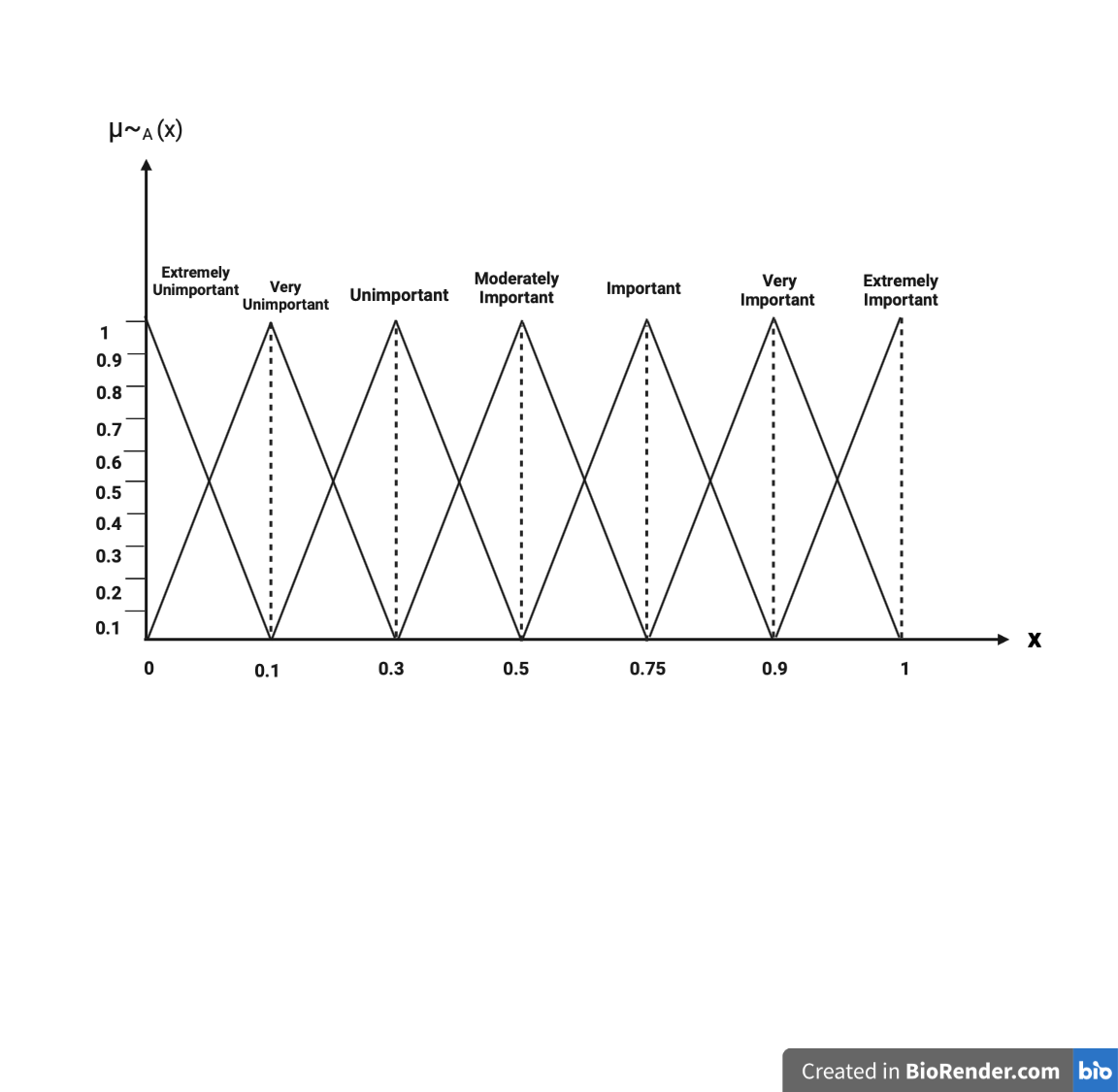


**Figure. S-2** The Fuzzy numbers Scale

Supplement: Multimedia component 3 [file mmc3.docx]

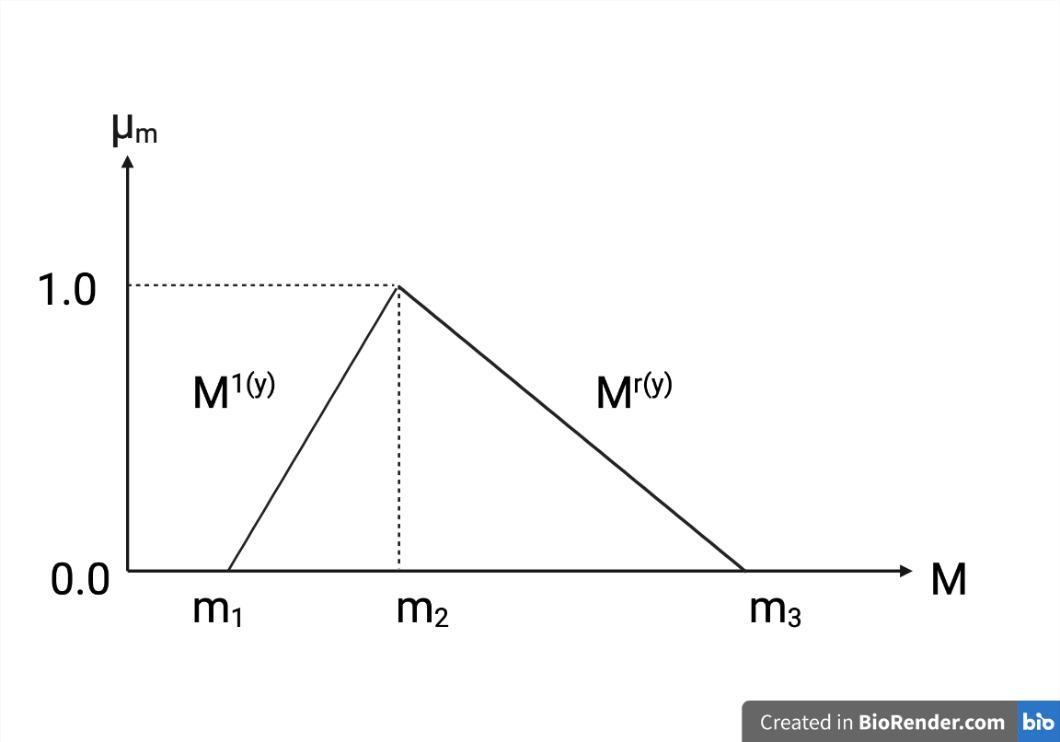


**Figure. S-3** Triangular Fuzzy Numbers

Supplement: Multimedia component 4 [file mmc4.docx]
