# Supplementary material for: Municipal solid waste management: Identification and analysis of technology selection criteria using Fuzzy Delphi and Fuzzy DEMATEL technique
Source: Heliyon. 2023 Dec 5;10(1):e23236. doi: 10.1016/j.heliyon.2023.e23236 (PMC10754890; doi:10.1016/j.heliyon.2023.e23236)
Supplement: Multimedia component 5 [file mmc5.docx]

**Table S-1** Information of the expert teams for FDM section

| **Expert** | **Expert category** | **Work description** | **Types of Organization** | **Number of personnel** | **Average working yr** |
| --- | --- | --- | --- | --- | --- |
| E_1_ | Researchers | Researchers from academia doing research in Waste management | Educational Institution,  Waste management projects | 5 | 6 |
| E_2_ |  |  |  |  |  |
| E_3_ |  |  |  |  |  |
| E_4_ |  |  |  |  |  |
| E_5_ |  |  |  |  |  |
| E_6_ | Municipal officials | Participating generally in developing and evaluating the policies and programs of the municipality | Gov’t municipal corporation | 3 | 11 |
| E_7_ |  |  |  |  |  |
| E_8_ |  |  |  |  |  |
| E_9_ | Waste Consultants | Specialists in waste management who are handling waste management projects from government and private firm as well | Waste management  Consultancy Firm | 2 | 7 |
| E_10_ |  |  |  |  |  |

**Table S-2** Fuzzy Delphi method (FDM) results

| **Criteria** | | **Condition of Triangular Fuzzy Numbers** | | **Condition of Defuzzification Process** | **Position/Rank** | **Experts Consensus** |
| --- | --- | --- | --- | --- | --- | --- |
|  |  | **Threshold Value, d**$\boldsymbol{\leq}$**0.2** | **Percentage of Experts Consensus,%>75%** | **Fuzzy Score (A) ≥ α–cut value** |  |  |
| **Technical** | **C_1_** | 0.000 | 100 % | 0.967 | 2 | Accepted |
|  | **C_2_** | 0.032 | 100 % | 0.960 | 15 | Accepted |
|  | **C_3_** | 0.042 | 100 % | 0.950 | 16 | Accepted |
|  | **C_4_** | 0.000 | 100 % | 0.967 | 2 | Accepted |
|  | **C_5_** | 0.183 | 50 % | 0.492 | 23 | Rejected |
|  | **C_6_** | 0.000 | 100 % | 0.967 | 2 | Accepted |
|  | **C_7_** | 0.042 | 100 % | 0.950 | 16 | Accepted |
|  | **C_8_** | 0.000 | 100 % | 0.989 | 1 | Accepted |
|  | **C_9_** | 0.000 | 100 % | 0.967 | 2 | Accepted |
| **Environmental** | **C_10_** | 0.056 | 100 % | 0.942 | 18 | Accepted |
|  | **C_11_** | 0.000 | 100 % | 0.967 | 2 | Accepted |
|  | **C_12_** | 0.163 | 60 % | 0.387 | 26 | Rejected |
|  | **C_13_** | 0.000 | 100 % | 0.967 | 2 | Accepted |
|  | **C_14_** | 0.000 | 100 % | 0.967 | 2 | Accepted |
|  | **C_15_** | 0.000 | 100 % | 0.967 | 2 | Accepted |
|  | **C_16_** | 0.163 | 50 % | 0.430 | 25 | Rejected |
| **Economical** | **C_17_** | 0.000 | 100 % | 0.967 | 2 | Accepted |
|  | **C_18_** | 0.000 | 100 % | 0.967 | 2 | Accepted |
|  | **C_19_** | 0.000 | 100 % | 0.967 | 2 | Accepted |
|  | **C_20_** | 0.183 | 50 % | 0.512 | 22 | Rejected |
|  | **C_21_** | 0.000 | 100 % | 0.967 | 2 | Accepted |
|  | **C_22_** | 0.000 | 100 % | 0.967 | 2 | Accepted |
|  | **C_23_** | 0.056 | 100 % | 0.908 | 20 | Accepted |
| **Social** | **C_24_** | 0.097 | 100 % | 0.833 | 21 | Accepted |
|  | **C_25_** | 0.143 | 70 % | 0.452 | 24 | Rejected |
|  | **C_26_** | 0.066 | 100 % | 0.925 | 19 | Accepted |

**Table S-3** Information of the expert teams

| **Expert** | **Expert category** | **Work description** | **Types of Organization** | **Number of personnel** | **Average working year** |
| --- | --- | --- | --- | --- | --- |
| E_1_ | Researchers | Researchers from Academia and different projects doing research in Waste management | Educational Institution,  Waste management Projects | 5 | 12 |
| E_2_ |  |  |  |  |  |
| E_3_ |  |  |  |  |  |
| E_4_ |  |  |  |  |  |
| E_5_ |  |  |  |  |  |
| E_6_ | Municipal officials | Participating generally in developing and evaluating the policies and programs of the municipality | Gov’t municipal corporation | 3 | 12 |
| E_7_ |  |  |  |  |  |
| E_8_ |  |  |  |  |  |
| E_9_ | Waste Consultants | Specialists in waste management who are handling waste management projects from government and private firm as well | Consultancy Firm | 3 | 11 |
| E_10_ |  |  |  |  |  |
| E_11_ |  |  |  |  |  |

**Table S-4** Decision matrix or Direct relation matrix, A

| **Criteria** | **T_1_** | **T_2_** | **T_3_** | **T_4_** | **T_5_** | **T_6_** | **T_7_** | **T_8_** | **E_1_** | **E_2_** | **E_3_** | **E_4_** | **E_5_** | **Ec_1_** | **Ec_2_** | **Ec_3_** | **Ec_4_** | **Ec_5_** | **Ec_6_** | **S_1_** | **S_2_** | **Row** |
| --- | --- | --- | --- | --- | --- | --- | --- | --- | --- | --- | --- | --- | --- | --- | --- | --- | --- | --- | --- | --- | --- | --- |
| **T_1_** | 0.8750 | 0.6620 | 0.7115 | 0.0000 | 0.1148 | 0.1698 | 0.2240 | 0.6390 | 0.5394 | 0.3969 | 0.2728 | 0.3433 | 0.2728 | 0.6144 | 0.2920 | 0.6962 | 0.1186 | 0.7647 | 0.0000 | 0.0000 | 0.0000 | 7.7072 |
| **T_2_** | 0.0000 | 0.8750 | 0.6811 | 0.0000 | 0.2703 | 0.2903 | 0.2378 | 0.5513 | 0.5936 | 0.3963 | 0.2553 | 0.3433 | 0.2553 | 0.6998 | 0.0343 | 0.0000 | 0.2728 | 0.4156 | 0.7133 | 0.3777 | 0.3777 | 7.6410 |
| **T_3_** | 0.6449 | 0.7476 | 0.8750 | 0.0000 | 0.3571 | 0.2728 | 0.2553 | 0.4671 | 0.4502 | 0.7647 | 0.5936 | 0.6439 | 0.5936 | 0.5858 | 0.0525 | 0.7133 | 0.5023 | 0.6620 | 0.6107 | 0.4671 | 0.4315 | 10.6910 |
| **T_4_** | 0.6278 | 0.7476 | 0.7476 | 0.8750 | 0.5556 | 0.4680 | 0.6791 | 0.6278 | 0.6107 | 0.7305 | 0.5584 | 0.5765 | 0.5765 | 0.6998 | 0.5584 | 0.6278 | 0.2203 | 0.5936 | 0.6620 | 0.5765 | 0.3800 | 12.6996 |
| **T_5_** | 0.0000 | 0.6107 | 0.7476 | 0.0000 | 0.8369 | 0.2553 | 0.6620 | 0.0000 | 0.0000 | 0.3078 | 0.0000 | 0.2378 | 0.2378 | 0.6998 | 0.0343 | 0.5917 | 0.0000 | 0.0000 | 0.1854 | 0.5765 | 0.3789 | 6.3624 |
| **T_6_** | 0.0000 | 0.6449 | 0.7286 | 0.0000 | 0.3395 | 0.8750 | 0.7647 | 0.2728 | 0.2728 | 0.2553 | 0.2553 | 0.2553 | 0.2553 | 0.6998 | 0.0525 | 0.6278 | 0.1854 | 0.5403 | 0.3078 | 0.5765 | 0.5223 | 8.4319 |
| **T_7_** | 0.2216 | 0.7305 | 0.7305 | 0.0000 | 0.5887 | 0.5042 | 0.8750 | 0.0000 | 0.0000 | 0.4867 | 0.3608 | 0.2909 | 0.3783 | 0.6998 | 0.0673 | 0.7647 | 0.5032 | 0.3801 | 0.4692 | 0.0000 | 0.0000 | 8.0514 |
| **T_8_** | 0.2728 | 0.5403 | 0.5765 | 0.0169 | 0.5727 | 0.4680 | 0.2915 | 0.8591 | 0.3078 | 0.5765 | 0.5765 | 0.5042 | 0.5765 | 0.6520 | 0.5585 | 0.0000 | 0.0000 | 0.0000 | 0.6107 | 0.0169 | 0.0524 | 8.0297 |
| **E_1_** | 0.0000 | 0.0337 | 0.5737 | 0.0000 | 0.2327 | 0.2029 | 0.0000 | 0.5032 | 0.8750 | 0.5936 | 0.5936 | 0.5936 | 0.5936 | 0.3756 | 0.5584 | 0.0000 | 0.0000 | 0.0000 | 0.5403 | 0.5765 | 0.2909 | 7.1373 |
| **E_2_** | 0.0000 | 0.0000 | 0.5737 | 0.0000 | 0.3752 | 0.2915 | 0.2029 | 0.0000 | 0.1180 | 0.8750 | 0.3078 | 0.6278 | 0.0337 | 0.1792 | 0.0168 | 0.3608 | 0.0000 | 0.3777 | 0.5765 | 0.2903 | 0.0000 | 5.2065 |
| **E_3_** | 0.0000 | 0.0000 | 0.5908 | 0.0000 | 0.5376 | 0.3608 | 0.3078 | 0.0000 | 0.0000 | 0.3602 | 0.8750 | 0.5765 | 0.5765 | 0.4460 | 0.0343 | 0.5403 | 0.0000 | 0.3777 | 0.5765 | 0.5765 | 0.5765 | 7.3128 |
| **E_4_** | 0.0000 | 0.0000 | 0.6928 | 0.0000 | 0.5727 | 0.3427 | 0.2029 | 0.0000 | 0.0000 | 0.7647 | 0.1854 | 0.8750 | 0.1854 | 0.2673 | 0.7294 | 0.6449 | 0.2391 | 0.3427 | 0.7647 | 0.2553 | 0.5765 | 7.6415 |
| **E_5_** | 0.0000 | 0.0000 | 0.6763 | 0.0000 | 0.5376 | 0.2909 | 0.1685 | 0.0169 | 0.0000 | 0.2222 | 0.7133 | 0.1348 | 0.8750 | 0.4845 | 0.7477 | 0.5042 | 0.2384 | 0.3427 | 0.5765 | 0.5765 | 0.5765 | 7.6824 |
| **Ec_1_** | 0.0000 | 0.0000 | 0.0000 | 0.0000 | 0.5013 | 0.4138 | 0.0000 | 0.0000 | 0.0000 | 0.0000 | 0.0000 | 0.0000 | 0.0000 | 0.7789 | 0.0343 | 0.0000 | 0.0000 | 0.0000 | 0.0000 | 0.0000 | 0.0000 | 1.7283 |
| **Ec_2_** | 0.3777 | 0.6278 | 0.7457 | 0.0000 | 0.4833 | 0.4138 | 0.5765 | 0.0000 | 0.0000 | 0.5765 | 0.3078 | 0.3078 | 0.3078 | 0.6998 | 0.8297 | 0.5936 | 0.5032 | 0.5574 | 0.5765 | 0.0000 | 0.0000 | 8.4849 |
| **Ec_3_** | 0.0000 | 0.6107 | 0.7628 | 0.0000 | 0.7230 | 0.5751 | 0.0000 | 0.0000 | 0.0000 | 0.0000 | 0.0000 | 0.0000 | 0.0000 | 0.6998 | 0.2384 | 0.8750 | 0.0000 | 0.0000 | 0.0000 | 0.0000 | 0.0000 | 4.4849 |
| **Ec_4_** | 0.0000 | 0.0000 | 0.5737 | 0.0000 | 0.6560 | 0.1186 | 0.0169 | 0.0000 | 0.0000 | 0.0000 | 0.0000 | 0.0000 | 0.0000 | 0.6998 | 0.3601 | 0.7647 | 0.8750 | 0.0674 | 0.0000 | 0.0000 | 0.0000 | 4.1322 |
| **Ec_5_** | 0.7647 | 0.0000 | 0.6250 | 0.0000 | 0.6731 | 0.0512 | 0.0000 | 0.0000 | 0.0000 | 0.0000 | 0.0000 | 0.0000 | 0.0000 | 0.6998 | 0.2061 | 0.7647 | 0.0000 | 0.8750 | 0.0000 | 0.0000 | 0.0000 | 4.6595 |
| **Ec_6_** | 0.4867 | 0.0000 | 0.6763 | 0.7647 | 0.6888 | 0.6449 | 0.7647 | 0.5765 | 0.5765 | 0.7647 | 0.5765 | 0.5765 | 0.5765 | 0.6998 | 0.7119 | 0.5936 | 0.0000 | 0.6962 | 0.8750 | 0.7647 | 0.5765 | 12.5909 |
| **S_1_** | 0.0000 | 0.5765 | 0.6944 | 0.0000 | 0.5556 | 0.0000 | 0.3957 | 0.0000 | 0.0000 | 0.0000 | 0.0000 | 0.0000 | 0.0000 | 0.2310 | 0.2560 | 0.0000 | 0.0000 | 0.3427 | 0.5765 | 0.8750 | 0.3078 | 4.8111 |
| **S_2_** | 0.0000 | 0.1854 | 0.3210 | 0.0000 | 0.7401 | 0.7476 | 0.3427 | 0.0000 | 0.0000 | 0.7476 | 0.0000 | 0.6449 | 0.0000 | 0.1428 | 0.0343 | 0.0000 | 0.0000 | 0.3602 | 0.7647 | 0.6449 | 0.8750 | 6.5512 |
|  |  |  |  |  |  |  |  |  |  |  |  |  |  |  |  |  |  |  |  | Max (K) | | 12.6996 |

**Table S-5** Normalized Direct relation matrix (X= K-A)

| **Criteria** | **T_1_** | **T_2_** | **T_3_** | **T_4_** | **T_5_** | **T_6_** | **T_7_** | **T_8_** | **E_1_** | **E_2_** | **E_3_** | **E_4_** | **E_5_** | **Ec_1_** | **Ec_2_** | **Ec_3_** | **Ec_4_** | **Ec_5_** | **Ec_6_** | **S_1_** | **S_2_** |
| --- | --- | --- | --- | --- | --- | --- | --- | --- | --- | --- | --- | --- | --- | --- | --- | --- | --- | --- | --- | --- | --- |
| **T_1_** | 0.0689 | 0.0521 | 0.0560 | 0.0000 | 0.0090 | 0.0134 | 0.0176 | 0.0503 | 0.0425 | 0.0313 | 0.0215 | 0.3433 | 0.2728 | 0.6144 | 0.2920 | 0.6962 | 0.1186 | 0.7647 | 0.0000 | 0.0000 | 0.0000 |
| **T_2_** | 0.0000 | 0.0689 | 0.0536 | 0.0000 | 0.0213 | 0.0229 | 0.0187 | 0.0434 | 0.0467 | 0.0312 | 0.0201 | 0.3433 | 0.2553 | 0.6998 | 0.0343 | 0.0000 | 0.2728 | 0.4156 | 0.7133 | 0.3777 | 0.3777 |
| **T_3_** | 0.0508 | 0.0589 | 0.0689 | 0.0000 | 0.0281 | 0.0215 | 0.0201 | 0.0368 | 0.0354 | 0.0602 | 0.0467 | 0.6439 | 0.5936 | 0.5858 | 0.0525 | 0.7133 | 0.5023 | 0.6620 | 0.6107 | 0.4671 | 0.4315 |
| **T_4_** | 0.0494 | 0.0589 | 0.0589 | 0.0689 | 0.0438 | 0.0369 | 0.0535 | 0.0494 | 0.0481 | 0.0575 | 0.0440 | 0.5765 | 0.5765 | 0.6998 | 0.5584 | 0.6278 | 0.2203 | 0.5936 | 0.6620 | 0.5765 | 0.3800 |
| **T_5_** | 0.0000 | 0.0481 | 0.0589 | 0.0000 | 0.0659 | 0.0201 | 0.0521 | 0.0000 | 0.0000 | 0.0242 | 0.0000 | 0.2378 | 0.2378 | 0.6998 | 0.0343 | 0.5917 | 0.0000 | 0.0000 | 0.1854 | 0.5765 | 0.3789 |
| **T_6_** | 0.0000 | 0.0508 | 0.0574 | 0.0000 | 0.0267 | 0.0689 | 0.0602 | 0.0215 | 0.0215 | 0.0201 | 0.0201 | 0.2553 | 0.2553 | 0.6998 | 0.0525 | 0.6278 | 0.1854 | 0.5403 | 0.3078 | 0.5765 | 0.5223 |
| **T_7_** | 0.0174 | 0.0575 | 0.0575 | 0.0000 | 0.0464 | 0.0397 | 0.0689 | 0.0000 | 0.0000 | 0.0383 | 0.0284 | 0.2909 | 0.3783 | 0.6998 | 0.0673 | 0.7647 | 0.5032 | 0.3801 | 0.4692 | 0.0000 | 0.0000 |
| **T_8_** | 0.0215 | 0.0425 | 0.0454 | 0.0013 | 0.0451 | 0.0369 | 0.0230 | 0.0676 | 0.0242 | 0.0454 | 0.0454 | 0.5042 | 0.5765 | 0.6520 | 0.5585 | 0.0000 | 0.0000 | 0.0000 | 0.6107 | 0.0169 | 0.0524 |
| **E_1_** | 0.0000 | 0.0027 | 0.0452 | 0.0000 | 0.0183 | 0.0160 | 0.0000 | 0.0396 | 0.0689 | 0.0467 | 0.0467 | 0.5936 | 0.5936 | 0.3756 | 0.5584 | 0.0000 | 0.0000 | 0.0000 | 0.5403 | 0.5765 | 0.2909 |
| **E_2_** | 0.0000 | 0.0000 | 0.0452 | 0.0000 | 0.0295 | 0.0230 | 0.0160 | 0.0000 | 0.0093 | 0.0689 | 0.0242 | 0.6278 | 0.0337 | 0.1792 | 0.0168 | 0.3608 | 0.0000 | 0.3777 | 0.5765 | 0.2903 | 0.0000 |
| **E_3_** | 0.0000 | 0.0000 | 0.0465 | 0.0000 | 0.0423 | 0.0284 | 0.0242 | 0.0000 | 0.0000 | 0.0284 | 0.0689 | 0.5765 | 0.5765 | 0.4460 | 0.0343 | 0.5403 | 0.0000 | 0.3777 | 0.5765 | 0.5765 | 0.5765 |
| **E_4_** | 0.0000 | 0.0000 | 0.0546 | 0.0000 | 0.0451 | 0.0270 | 0.0160 | 0.0000 | 0.0000 | 0.0602 | 0.0146 | 0.8750 | 0.1854 | 0.2673 | 0.7294 | 0.6449 | 0.2391 | 0.3427 | 0.7647 | 0.2553 | 0.5765 |
| **E_5_** | 0.0000 | 0.0000 | 0.0533 | 0.0000 | 0.0423 | 0.0229 | 0.0133 | 0.0013 | 0.0000 | 0.0175 | 0.0562 | 0.1348 | 0.8750 | 0.4845 | 0.7477 | 0.5042 | 0.2384 | 0.3427 | 0.5765 | 0.5765 | 0.5765 |
| **Ec_1_** | 0.0000 | 0.0000 | 0.0000 | 0.0000 | 0.0395 | 0.0326 | 0.0000 | 0.0000 | 0.0000 | 0.0000 | 0.0000 | 0.0000 | 0.0000 | 0.7789 | 0.0343 | 0.0000 | 0.0000 | 0.0000 | 0.0000 | 0.0000 | 0.0000 |
| **Ec_2_** | 0.0297 | 0.0494 | 0.0587 | 0.0000 | 0.0381 | 0.0326 | 0.0454 | 0.0000 | 0.0000 | 0.0454 | 0.0242 | 0.3078 | 0.3078 | 0.6998 | 0.8297 | 0.5936 | 0.5032 | 0.5574 | 0.5765 | 0.0000 | 0.0000 |
| **Ec_3_** | 0.0000 | 0.0481 | 0.0601 | 0.0000 | 0.0569 | 0.0453 | 0.0000 | 0.0000 | 0.0000 | 0.0000 | 0.0000 | 0.0000 | 0.0000 | 0.6998 | 0.2384 | 0.8750 | 0.0000 | 0.0000 | 0.0000 | 0.0000 | 0.0000 |
| **Ec_4_** | 0.0000 | 0.0000 | 0.0452 | 0.0000 | 0.0517 | 0.0093 | 0.0013 | 0.0000 | 0.0000 | 0.0000 | 0.0000 | 0.0000 | 0.0000 | 0.6998 | 0.3601 | 0.7647 | 0.8750 | 0.0674 | 0.0000 | 0.0000 | 0.0000 |
| **Ec_5_** | 0.0602 | 0.0000 | 0.0492 | 0.0000 | 0.0530 | 0.0040 | 0.0000 | 0.0000 | 0.0000 | 0.0000 | 0.0000 | 0.0000 | 0.0000 | 0.6998 | 0.2061 | 0.7647 | 0.0000 | 0.8750 | 0.0000 | 0.0000 | 0.0000 |
| **Ec_6_** | 0.0383 | 0.0000 | 0.0533 | 0.0602 | 0.0542 | 0.0508 | 0.0602 | 0.0454 | 0.0454 | 0.0602 | 0.0454 | 0.5765 | 0.5765 | 0.6998 | 0.7119 | 0.5936 | 0.0000 | 0.6962 | 0.8750 | 0.7647 | 0.5765 |
| **S_1_** | 0.0000 | 0.0454 | 0.0547 | 0.0000 | 0.0438 | 0.0000 | 0.0312 | 0.0000 | 0.0000 | 0.0000 | 0.0000 | 0.0000 | 0.0000 | 0.2310 | 0.2560 | 0.0000 | 0.0000 | 0.3427 | 0.5765 | 0.8750 | 0.3078 |
| **S_2_** | 0.0000 | 0.0146 | 0.0253 | 0.0000 | 0.0583 | 0.0589 | 0.0270 | 0.0000 | 0.0000 | 0.0589 | 0.0000 | 0.6449 | 0.0000 | 0.1428 | 0.0343 | 0.0000 | 0.0000 | 0.3602 | 0.7647 | 0.6449 | 0.8750 |

**Table S-6** Identity matrix, I

| **Criteria** | **T_1_** | **T_2_** | **T_3_** | **T_4_** | **T_5_** | **T_6_** | **T_7_** | **T_8_** | **E_1_** | **E_2_** | **E_3_** | **E_4_** | **E_5_** | **Ec_1_** | **Ec_2_** | **Ec_3_** | **Ec_4_** | **Ec_5_** | **Ec_6_** | **S_1_** | **S_2_** |
| --- | --- | --- | --- | --- | --- | --- | --- | --- | --- | --- | --- | --- | --- | --- | --- | --- | --- | --- | --- | --- | --- |
| **T_1_** | 1.0000 | 0.0000 | 0.0000 | 0.0000 | 0.0000 | 0.0000 | 0.0000 | 0.0000 | 0.0000 | 0.0000 | 0.0000 | 0.0000 | 0.0000 | 0.0000 | 0.0000 | 0.0000 | 0.0000 | 0.0000 | 0.0000 | 0.0000 | 0.0000 |
| **T_2_** | 0.0000 | 1.0000 | 0.0000 | 0.0000 | 0.0000 | 0.0000 | 0.0000 | 0.0000 | 0.0000 | 0.0000 | 0.0000 | 0.0000 | 0.0000 | 0.0000 | 0.0000 | 0.0000 | 0.0000 | 0.0000 | 0.0000 | 0.0000 | 0.0000 |
| **T_3_** | 0.0000 | 0.0000 | 1.0000 | 0.0000 | 0.0000 | 0.0000 | 0.0000 | 0.0000 | 0.0000 | 0.0000 | 0.0000 | 0.0000 | 0.0000 | 0.0000 | 0.0000 | 0.0000 | 0.0000 | 0.0000 | 0.0000 | 0.0000 | 0.0000 |
| **T_4_** | 0.0000 | 0.0000 | 0.0000 | 1.0000 | 0.0000 | 0.0000 | 0.0000 | 0.0000 | 0.0000 | 0.0000 | 0.0000 | 0.0000 | 0.0000 | 0.0000 | 0.0000 | 0.0000 | 0.0000 | 0.0000 | 0.0000 | 0.0000 | 0.0000 |
| **T_5_** | 0.0000 | 0.0000 | 0.0000 | 0.0000 | 1.0000 | 0.0000 | 0.0000 | 0.0000 | 0.0000 | 0.0000 | 0.0000 | 0.0000 | 0.0000 | 0.0000 | 0.0000 | 0.0000 | 0.0000 | 0.0000 | 0.0000 | 0.0000 | 0.0000 |
| **T_6_** | 0.0000 | 0.0000 | 0.0000 | 0.0000 | 0.0000 | 1.0000 | 0.0000 | 0.0000 | 0.0000 | 0.0000 | 0.0000 | 0.0000 | 0.0000 | 0.0000 | 0.0000 | 0.0000 | 0.0000 | 0.0000 | 0.0000 | 0.0000 | 0.0000 |
| **T_7_** | 0.0000 | 0.0000 | 0.0000 | 0.0000 | 0.0000 | 0.0000 | 1.0000 | 0.0000 | 0.0000 | 0.0000 | 0.0000 | 0.0000 | 0.0000 | 0.0000 | 0.0000 | 0.0000 | 0.0000 | 0.0000 | 0.0000 | 0.0000 | 0.0000 |
| **T_8_** | 0.0000 | 0.0000 | 0.0000 | 0.0000 | 0.0000 | 0.0000 | 0.0000 | 1.0000 | 0.0000 | 0.0000 | 0.0000 | 0.0000 | 0.0000 | 0.0000 | 0.0000 | 0.0000 | 0.0000 | 0.0000 | 0.0000 | 0.0000 | 0.0000 |
| **E_1_** | 0.0000 | 0.0000 | 0.0000 | 0.0000 | 0.0000 | 0.0000 | 0.0000 | 0.0000 | 1.0000 | 0.0000 | 0.0000 | 0.0000 | 0.0000 | 0.0000 | 0.0000 | 0.0000 | 0.0000 | 0.0000 | 0.0000 | 0.0000 | 0.0000 |
| **E_2_** | 0.0000 | 0.0000 | 0.0000 | 0.0000 | 0.0000 | 0.0000 | 0.0000 | 0.0000 | 0.0000 | 1.0000 | 0.0000 | 0.0000 | 0.0000 | 0.0000 | 0.0000 | 0.0000 | 0.0000 | 0.0000 | 0.0000 | 0.0000 | 0.0000 |
| **E_3_** | 0.0000 | 0.0000 | 0.0000 | 0.0000 | 0.0000 | 0.0000 | 0.0000 | 0.0000 | 0.0000 | 0.0000 | 1.0000 | 0.0000 | 0.0000 | 0.0000 | 0.0000 | 0.0000 | 0.0000 | 0.0000 | 0.0000 | 0.0000 | 0.0000 |
| **E_4_** | 0.0000 | 0.0000 | 0.0000 | 0.0000 | 0.0000 | 0.0000 | 0.0000 | 0.0000 | 0.0000 | 0.0000 | 0.0000 | 1.0000 | 0.0000 | 0.0000 | 0.0000 | 0.0000 | 0.0000 | 0.0000 | 0.0000 | 0.0000 | 0.0000 |
| **E_5_** | 0.0000 | 0.0000 | 0.0000 | 0.0000 | 0.0000 | 0.0000 | 0.0000 | 0.0000 | 0.0000 | 0.0000 | 0.0000 | 0.0000 | 1.0000 | 0.0000 | 0.0000 | 0.0000 | 0.0000 | 0.0000 | 0.0000 | 0.0000 | 0.0000 |
| **Ec_1_** | 0.0000 | 0.0000 | 0.0000 | 0.0000 | 0.0000 | 0.0000 | 0.0000 | 0.0000 | 0.0000 | 0.0000 | 0.0000 | 0.0000 | 0.0000 | 1.0000 | 0.0000 | 0.0000 | 0.0000 | 0.0000 | 0.0000 | 0.0000 | 0.0000 |
| **Ec_2_** | 0.0000 | 0.0000 | 0.0000 | 0.0000 | 0.0000 | 0.0000 | 0.0000 | 0.0000 | 0.0000 | 0.0000 | 0.0000 | 0.0000 | 0.0000 | 0.0000 | 1.0000 | 0.0000 | 0.0000 | 0.0000 | 0.0000 | 0.0000 | 0.0000 |
| **Ec_3_** | 0.0000 | 0.0000 | 0.0000 | 0.0000 | 0.0000 | 0.0000 | 0.0000 | 0.0000 | 0.0000 | 0.0000 | 0.0000 | 0.0000 | 0.0000 | 0.0000 | 0.0000 | 1.0000 | 0.0000 | 0.0000 | 0.0000 | 0.0000 | 0.0000 |
| **Ec_4_** | 0.0000 | 0.0000 | 0.0000 | 0.0000 | 0.0000 | 0.0000 | 0.0000 | 0.0000 | 0.0000 | 0.0000 | 0.0000 | 0.0000 | 0.0000 | 0.0000 | 0.0000 | 0.0000 | 1.0000 | 0.0000 | 0.0000 | 0.0000 | 0.0000 |
| **Ec_5_** | 0.0000 | 0.0000 | 0.0000 | 0.0000 | 0.0000 | 0.0000 | 0.0000 | 0.0000 | 0.0000 | 0.0000 | 0.0000 | 0.0000 | 0.0000 | 0.0000 | 0.0000 | 0.0000 | 0.0000 | 1.0000 | 0.0000 | 0.0000 | 0.0000 |
| **Ec_6_** | 0.0000 | 0.0000 | 0.0000 | 0.0000 | 0.0000 | 0.0000 | 0.0000 | 0.0000 | 0.0000 | 0.0000 | 0.0000 | 0.0000 | 0.0000 | 0.0000 | 0.0000 | 0.0000 | 0.0000 | 0.0000 | 1.0000 | 0.0000 | 0.0000 |
| **S_1_** | 0.0000 | 0.0000 | 0.0000 | 0.0000 | 0.0000 | 0.0000 | 0.0000 | 0.0000 | 0.0000 | 0.0000 | 0.0000 | 0.0000 | 0.0000 | 0.0000 | 0.0000 | 0.0000 | 0.0000 | 0.0000 | 0.0000 | 1.0000 | 0.0000 |
| **S_2_** | 0.0000 | 0.0000 | 0.0000 | 0.0000 | 0.0000 | 0.0000 | 0.0000 | 0.0000 | 0.0000 | 0.0000 | 0.0000 | 0.0000 | 0.0000 | 0.0000 | 0.0000 | 0.0000 | 0.0000 | 0.0000 | 0.0000 | 0.0000 | 1.0000 |

**Table S-7** Identity matrix, I – Normalized Direct relation matrix, X

| **Criteria** | **T_1_** | **T_2_** | **T_3_** | **T_4_** | **T_5_** | **T_6_** | **T_7_** | **T_8_** | **E_1_** | **E_2_** | **E_3_** | **E_4_** | **E_5_** | **Ec_1_** | **Ec_2_** | **Ec_3_** | **Ec_4_** | **Ec_5_** | **Ec_6_** | **S_1_** | **S_2_** |
| --- | --- | --- | --- | --- | --- | --- | --- | --- | --- | --- | --- | --- | --- | --- | --- | --- | --- | --- | --- | --- | --- |
| **T_1_** | 0.9311 | (0.0521) | (0.0560) | 0.0000 | (0.0090) | (0.0134) | (0.0176) | (0.0503) | (0.0425) | (0.0313) | (0.0215) | (0.0270) | (0.0215) | (0.0484) | (0.0230) | (0.0548) | (0.0093) | (0.0602) | 0.0000 | 0.0000 | 0.0000 |
| **T_2_** | 0.0000 | 0.9311 | (0.0536) | 0.0000 | (0.0213) | (0.0229) | (0.0187) | (0.0434) | (0.0467) | (0.0312) | (0.0201) | (0.0270) | (0.0201) | (0.0551) | (0.0027) | 0.0000 | (0.0215) | (0.0327) | (0.0562) | (0.0297) | (0.0297) |
| **T_3_** | (0.0508) | (0.0589) | 0.9311 | 0.0000 | (0.0281) | (0.0215) | (0.0201) | (0.0368) | (0.0354) | (0.0602) | (0.0467) | (0.0507) | (0.0467) | (0.0461) | (0.0041) | (0.0562) | (0.0395) | (0.0521) | (0.0481) | (0.0368) | (0.0340) |
| **T_4_** | (0.0494) | (0.0589) | (0.0589) | 0.9311 | (0.0438) | (0.0369) | (0.0535) | (0.0494) | (0.0481) | (0.0575) | (0.0440) | (0.0454) | (0.0454) | (0.0551) | (0.0440) | (0.0494) | (0.0174) | (0.0467) | (0.0521) | (0.0454) | (0.0299) |
| **T_5_** | 0.0000 | (0.0481) | (0.0589) | 0.0000 | 0.9341 | (0.0201) | (0.0521) | 0.0000 | 0.0000 | (0.0242) | 0.0000 | (0.0187) | (0.0187) | (0.0551) | (0.0027) | (0.0466) | 0.0000 | 0.0000 | (0.0146) | (0.0454) | (0.0298) |
| **T_6_** | 0.0000 | (0.0508) | (0.0574) | 0.0000 | (0.0267) | 0.9311 | (0.0602) | (0.0215) | (0.0215) | (0.0201) | (0.0201) | (0.0201) | (0.0201) | (0.0551) | (0.0041) | (0.0494) | (0.0146) | (0.0425) | (0.0242) | (0.0454) | (0.0411) |
| **T_7_** | (0.0174) | (0.0575) | (0.0575) | 0.0000 | (0.0464) | (0.0397) | 0.9311 | 0.0000 | 0.0000 | (0.0383) | (0.0284) | (0.0229) | (0.0298) | (0.0551) | (0.0053) | (0.0602) | (0.0396) | (0.0299) | (0.0369) | 0.0000 | 0.0000 |
| **T_8_** | (0.0215) | (0.0425) | (0.0454) | (0.0013) | (0.0451) | (0.0369) | (0.0230) | 0.9324 | (0.0242) | (0.0454) | (0.0454) | (0.0397) | (0.0454) | (0.0513) | (0.0440) | 0.0000 | 0.0000 | 0.0000 | (0.0481) | (0.0013) | (0.0041) |
| **E_1_** | 0.0000 | (0.0027) | (0.0452) | 0.0000 | (0.0183) | (0.0160) | 0.0000 | (0.0396) | 0.9311 | (0.0467) | (0.0467) | (0.0467) | (0.0467) | (0.0296) | (0.0440) | 0.0000 | 0.0000 | 0.0000 | (0.0425) | (0.0454) | (0.0229) |
| **E_2_** | 0.0000 | 0.0000 | (0.0452) | 0.0000 | (0.0295) | (0.0230) | (0.0160) | 0.0000 | (0.0093) | 0.9311 | (0.0242) | (0.0494) | (0.0027) | (0.0141) | (0.0013) | (0.0284) | 0.0000 | (0.0297) | (0.0454) | (0.0229) | 0.0000 |
| **E_3_** | 0.0000 | 0.0000 | (0.0465) | 0.0000 | (0.0423) | (0.0284) | (0.0242) | 0.0000 | 0.0000 | (0.0284) | 0.9311 | (0.0454) | (0.0454) | (0.0351) | (0.0027) | (0.0425) | 0.0000 | (0.0297) | (0.0454) | (0.0454) | (0.0454) |
| **E_4_** | 0.0000 | 0.0000 | (0.0546) | 0.0000 | (0.0451) | (0.0270) | (0.0160) | 0.0000 | 0.0000 | (0.0602) | (0.0146) | 0.9311 | (0.0146) | (0.0211) | (0.0574) | (0.0508) | (0.0188) | (0.0270) | (0.0602) | (0.0201) | (0.0454) |
| **E_5_** | 0.0000 | 0.0000 | (0.0533) | 0.0000 | (0.0423) | (0.0229) | (0.0133) | (0.0013) | 0.0000 | (0.0175) | (0.0562) | (0.0106) | 0.9311 | (0.0382) | (0.0589) | (0.0397) | (0.0188) | (0.0270) | (0.0454) | (0.0454) | (0.0454) |
| **Ec_1_** | 0.0000 | 0.0000 | 0.0000 | 0.0000 | (0.0395) | (0.0326) | 0.0000 | 0.0000 | 0.0000 | 0.0000 | 0.0000 | 0.0000 | 0.0000 | 0.9387 | (0.0027) | 0.0000 | 0.0000 | 0.0000 | 0.0000 | 0.0000 | 0.0000 |
| **Ec_2_** | (0.0297) | (0.0494) | (0.0587) | 0.0000 | (0.0381) | (0.0326) | (0.0454) | 0.0000 | 0.0000 | (0.0454) | (0.0242) | (0.0242) | (0.0242) | (0.0551) | 0.9347 | (0.0467) | (0.0396) | (0.0439) | (0.0454) | 0.0000 | 0.0000 |
| **Ec_3_** | 0.0000 | (0.0481) | (0.0601) | 0.0000 | (0.0569) | (0.0453) | 0.0000 | 0.0000 | 0.0000 | 0.0000 | 0.0000 | 0.0000 | 0.0000 | (0.0551) | (0.0188) | 0.9311 | 0.0000 | 0.0000 | 0.0000 | 0.0000 | 0.0000 |
| **Ec_4_** | 0.0000 | 0.0000 | (0.0452) | 0.0000 | (0.0517) | (0.0093) | (0.0013) | 0.0000 | 0.0000 | 0.0000 | 0.0000 | 0.0000 | 0.0000 | (0.0551) | (0.0284) | (0.0602) | 0.9311 | (0.0053) | 0.0000 | 0.0000 | 0.0000 |
| **Ec_5_** | (0.0602) | 0.0000 | (0.0492) | 0.0000 | (0.0530) | (0.0040) | 0.0000 | 0.0000 | 0.0000 | 0.0000 | 0.0000 | 0.0000 | 0.0000 | (0.0551) | (0.0162) | (0.0602) | 0.0000 | 0.9311 | 0.0000 | 0.0000 | 0.0000 |
| **Ec_6_** | (0.0383) | 0.0000 | (0.0533) | (0.0602) | (0.0542) | (0.0508) | (0.0602) | (0.0454) | (0.0454) | (0.0602) | (0.0454) | (0.0454) | (0.0454) | (0.0551) | (0.0561) | (0.0467) | 0.0000 | (0.0548) | 0.9311 | (0.0602) | (0.0454) |
| **S_1_** | 0.0000 | (0.0454) | (0.0547) | 0.0000 | (0.0438) | 0.0000 | (0.0312) | 0.0000 | 0.0000 | 0.0000 | 0.0000 | 0.0000 | 0.0000 | (0.0182) | (0.0202) | 0.0000 | 0.0000 | (0.0270) | (0.0454) | 0.9311 | (0.0242) |
| **S_2_** | 0.0000 | (0.0146) | (0.0253) | 0.0000 | (0.0583) | (0.0589) | (0.0270) | 0.0000 | 0.0000 | (0.0589) | 0.0000 | (0.0508) | 0.0000 | (0.0112) | (0.0027) | 0.0000 | 0.0000 | (0.0284) | (0.0602) | (0.0508) | 0.9311 |

**Table S-8** Inverse matrix of I-X, (I−X)−1

| **Criteria** | **T_1_** | **T_2_** | **T_3_** | **T_4_** | **T_5_** | **T_6_** | **T_7_** | **T_8_** | **E_1_** | **E_2_** | **E_3_** | **E_4_** | **E_5_** | **Ec_1_** | **Ec_2_** | **Ec_3_** | **Ec_4_** | **Ec_5_** | **Ec_6_** | **S_1_** | **S_2_** |
| --- | --- | --- | --- | --- | --- | --- | --- | --- | --- | --- | --- | --- | --- | --- | --- | --- | --- | --- | --- | --- | --- |
| **T_1_** | 1.0929 | 0.0909 | 0.1211 | 0.0027 | 0.0600 | 0.0495 | 0.0449 | 0.0741 | 0.0649 | 0.0723 | 0.0519 | 0.0625 | 0.0510 | 0.1089 | 0.0503 | 0.1031 | 0.0254 | 0.0982 | 0.0407 | 0.0285 | 0.0242 |
| **T_2_** | 0.0194 | 1.1053 | 0.1196 | 0.0070 | 0.0771 | 0.0627 | 0.0528 | 0.0673 | 0.0705 | 0.0775 | 0.0527 | 0.0668 | 0.0519 | 0.1153 | 0.0312 | 0.0440 | 0.0379 | 0.0712 | 0.1070 | 0.0695 | 0.0626 |
| **T_3_** | 0.0792 | 0.1089 | 1.1611 | 0.0073 | 0.1044 | 0.0743 | 0.0633 | 0.0655 | 0.0637 | 0.1203 | 0.0893 | 0.1027 | 0.0878 | 0.1276 | 0.0425 | 0.1241 | 0.0623 | 0.1057 | 0.1117 | 0.0868 | 0.0759 |
| **T_4_** | 0.0871 | 0.1270 | 0.1745 | 1.0828 | 0.1386 | 0.1048 | 0.1144 | 0.0876 | 0.0855 | 0.1350 | 0.0992 | 0.1110 | 0.0995 | 0.1586 | 0.0946 | 0.1338 | 0.0462 | 0.1145 | 0.1338 | 0.1084 | 0.0812 |
| **T_5_** | 0.0127 | 0.0841 | 0.1124 | 0.0033 | 1.1122 | 0.0526 | 0.0819 | 0.0136 | 0.0139 | 0.0577 | 0.0206 | 0.0464 | 0.0403 | 0.1038 | 0.0198 | 0.0847 | 0.0138 | 0.0282 | 0.0515 | 0.0765 | 0.0550 |
| **T_6_** | 0.0194 | 0.0951 | 0.1288 | 0.0048 | 0.0874 | 1.1138 | 0.0986 | 0.0419 | 0.0418 | 0.0641 | 0.0503 | 0.0575 | 0.0502 | 0.1206 | 0.0294 | 0.1013 | 0.0330 | 0.0835 | 0.0732 | 0.0855 | 0.0746 |
| **T_7_** | 0.0370 | 0.0991 | 0.1268 | 0.0052 | 0.1044 | 0.0814 | 1.1047 | 0.0186 | 0.0188 | 0.0800 | 0.0582 | 0.0579 | 0.0593 | 0.1206 | 0.0296 | 0.1157 | 0.0601 | 0.0686 | 0.0806 | 0.0342 | 0.0288 |
| **T_8_** | 0.0427 | 0.0837 | 0.1185 | 0.0082 | 0.1053 | 0.0820 | 0.0632 | 1.0929 | 0.0474 | 0.0977 | 0.0845 | 0.0844 | 0.0835 | 0.1178 | 0.0776 | 0.0517 | 0.0182 | 0.0415 | 0.1033 | 0.0420 | 0.0387 |
| **E_1_** | 0.0170 | 0.0342 | 0.1098 | 0.0062 | 0.0718 | 0.0540 | 0.0335 | 0.0594 | 1.0893 | 0.0943 | 0.0820 | 0.0880 | 0.0805 | 0.0832 | 0.0767 | 0.0433 | 0.0147 | 0.0363 | 0.0946 | 0.0864 | 0.0561 |
| **E_2_** | 0.0140 | 0.0236 | 0.0928 | 0.0051 | 0.0690 | 0.0503 | 0.0406 | 0.0118 | 0.0219 | 1.1021 | 0.0453 | 0.0780 | 0.0221 | 0.0535 | 0.0198 | 0.0657 | 0.0101 | 0.0581 | 0.0790 | 0.0499 | 0.0216 |
| **E_3_** | 0.0167 | 0.0330 | 0.1102 | 0.0059 | 0.0989 | 0.0676 | 0.0586 | 0.0138 | 0.0140 | 0.0694 | 1.0982 | 0.0815 | 0.0738 | 0.0896 | 0.0277 | 0.0914 | 0.0136 | 0.0671 | 0.0915 | 0.0854 | 0.0796 |
| **E_4_** | 0.0202 | 0.0368 | 0.1238 | 0.0071 | 0.1049 | 0.0695 | 0.0532 | 0.0157 | 0.0163 | 0.1085 | 0.0429 | 1.1093 | 0.0421 | 0.0802 | 0.0877 | 0.1056 | 0.0368 | 0.0678 | 0.1094 | 0.0578 | 0.0779 |
| **E_5_** | 0.0191 | 0.0364 | 0.1205 | 0.0060 | 0.1015 | 0.0635 | 0.0495 | 0.0160 | 0.0146 | 0.0592 | 0.0874 | 0.0456 | 1.1000 | 0.0979 | 0.0881 | 0.0913 | 0.0362 | 0.0667 | 0.0928 | 0.0856 | 0.0791 |
| **Ec_1_** | 0.0014 | 0.0071 | 0.0096 | 0.0003 | 0.0501 | 0.0411 | 0.0071 | 0.0021 | 0.0021 | 0.0049 | 0.0028 | 0.0041 | 0.0036 | 1.0743 | 0.0050 | 0.0074 | 0.0019 | 0.0043 | 0.0050 | 0.0063 | 0.0050 |
| **Ec_2_** | 0.0541 | 0.0930 | 0.1328 | 0.0060 | 0.0992 | 0.0761 | 0.0834 | 0.0200 | 0.0202 | 0.0915 | 0.0561 | 0.0622 | 0.0555 | 0.1250 | 1.0953 | 0.1064 | 0.0622 | 0.0881 | 0.0925 | 0.0347 | 0.0290 |
| **Ec_3_** | 0.0090 | 0.0762 | 0.0975 | 0.0014 | 0.0879 | 0.0694 | 0.0187 | 0.0111 | 0.0112 | 0.0205 | 0.0135 | 0.0172 | 0.0146 | 0.0925 | 0.0294 | 1.0970 | 0.0098 | 0.0183 | 0.0216 | 0.0191 | 0.0160 |
| **Ec_4_** | 0.0075 | 0.0194 | 0.0754 | 0.0009 | 0.0799 | 0.0272 | 0.0144 | 0.0059 | 0.0058 | 0.0143 | 0.0089 | 0.0116 | 0.0100 | 0.0872 | 0.0392 | 0.0870 | 1.0809 | 0.0180 | 0.0137 | 0.0121 | 0.0098 |
| **Ec_5_** | 0.0773 | 0.0238 | 0.0853 | 0.0010 | 0.0835 | 0.0232 | 0.0144 | 0.0104 | 0.0097 | 0.0178 | 0.0115 | 0.0148 | 0.0126 | 0.0920 | 0.0280 | 0.0917 | 0.0077 | 1.0909 | 0.0151 | 0.0134 | 0.0109 |
| **Ec_6_** | 0.0760 | 0.0655 | 0.1679 | 0.0745 | 0.1506 | 0.1199 | 0.1237 | 0.0800 | 0.0793 | 0.1374 | 0.0995 | 0.1104 | 0.0985 | 0.1566 | 0.1069 | 0.1322 | 0.0267 | 0.1229 | 1.1500 | 0.1246 | 0.0974 |
| **S_1_** | 0.0150 | 0.0748 | 0.0994 | 0.0051 | 0.0814 | 0.0241 | 0.0572 | 0.0134 | 0.0135 | 0.0282 | 0.0178 | 0.0229 | 0.0186 | 0.0576 | 0.0364 | 0.0299 | 0.0114 | 0.0546 | 0.0782 | 1.0969 | 0.0455 |
| **S_2_** | 0.0158 | 0.0473 | 0.0843 | 0.0068 | 0.1099 | 0.0965 | 0.0633 | 0.0148 | 0.0154 | 0.1013 | 0.0226 | 0.0866 | 0.0223 | 0.0600 | 0.0246 | 0.0424 | 0.0124 | 0.0649 | 0.1053 | 0.0894 | 1.1009 |

**Table S-9** Total Relation matrix (T)

| **Criteria** | **T_1_** | **T_2_** | **T_3_** | **T_4_** | **T_5_** | **T_6_** | **T_7_** | **T_8_** | **E_1_** | **E_2_** | **E_3_** | **E_4_** | **E_5_** | **Ec_1_** | **Ec_2_** | **Ec_3_** | **Ec_4_** | **Ec_5_** | **Ec_6_** | **S_1_** | **S_2_** | **D** |
| --- | --- | --- | --- | --- | --- | --- | --- | --- | --- | --- | --- | --- | --- | --- | --- | --- | --- | --- | --- | --- | --- | --- |
| **T_1_** | 0.0929 | 0.0909 | 0.1211 | 0.0027 | 0.0600 | 0.0495 | 0.0449 | 0.0741 | 0.0649 | 0.0723 | 0.0519 | 0.0625 | 0.0510 | 0.1089 | 0.0503 | 0.1031 | 0.0254 | 0.0982 | 0.0407 | 0.0285 | 0.0242 | **1.3182** |
| **T_2_** | 0.0194 | 0.1053 | 0.1196 | 0.0070 | 0.0771 | 0.0627 | 0.0528 | 0.0673 | 0.0705 | 0.0775 | 0.0527 | 0.0668 | 0.0519 | 0.1153 | 0.0312 | 0.0440 | 0.0379 | 0.0712 | 0.1070 | 0.0695 | 0.0626 | **1.3694** |
| **T_3_** | 0.0792 | 0.1089 | 0.1611 | 0.0073 | 0.1044 | 0.0743 | 0.0633 | 0.0655 | 0.0637 | 0.1203 | 0.0893 | 0.1027 | 0.0878 | 0.1276 | 0.0425 | 0.1241 | 0.0623 | 0.1057 | 0.1117 | 0.0868 | 0.0759 | **1.8644** |
| **T_4_** | 0.0871 | 0.1270 | 0.1745 | 0.0828 | 0.1386 | 0.1048 | 0.1144 | 0.0876 | 0.0855 | 0.1350 | 0.0992 | 0.1110 | 0.0995 | 0.1586 | 0.0946 | 0.1338 | 0.0462 | 0.1145 | 0.1338 | 0.1084 | 0.0812 | **2.3180** |
| **T_5_** | 0.0127 | 0.0841 | 0.1124 | 0.0033 | 0.1122 | 0.0526 | 0.0819 | 0.0136 | 0.0139 | 0.0577 | 0.0206 | 0.0464 | 0.0403 | 0.1038 | 0.0198 | 0.0847 | 0.0138 | 0.0282 | 0.0515 | 0.0765 | 0.0550 | **1.0851** |
| **T_6_** | 0.0194 | 0.0951 | 0.1288 | 0.0048 | 0.0874 | 0.1138 | 0.0986 | 0.0419 | 0.0418 | 0.0641 | 0.0503 | 0.0575 | 0.0502 | 0.1206 | 0.0294 | 0.1013 | 0.0330 | 0.0835 | 0.0732 | 0.0855 | 0.0746 | **1.4549** |
| **T_7_** | 0.0370 | 0.0991 | 0.1268 | 0.0052 | 0.1044 | 0.0814 | 0.1047 | 0.0186 | 0.0188 | 0.0800 | 0.0582 | 0.0579 | 0.0593 | 0.1206 | 0.0296 | 0.1157 | 0.0601 | 0.0686 | 0.0806 | 0.0342 | 0.0288 | **1.3896** |
| **T_8_** | 0.0427 | 0.0837 | 0.1185 | 0.0082 | 0.1053 | 0.0820 | 0.0632 | 0.0929 | 0.0474 | 0.0977 | 0.0845 | 0.0844 | 0.0835 | 0.1178 | 0.0776 | 0.0517 | 0.0182 | 0.0415 | 0.1033 | 0.0420 | 0.0387 | **1.4847** |
| **E_1_** | 0.0170 | 0.0342 | 0.1098 | 0.0062 | 0.0718 | 0.0540 | 0.0335 | 0.0594 | 0.0893 | 0.0943 | 0.0820 | 0.0880 | 0.0805 | 0.0832 | 0.0767 | 0.0433 | 0.0147 | 0.0363 | 0.0946 | 0.0864 | 0.0561 | **1.3112** |
| **E_2_** | 0.0140 | 0.0236 | 0.0928 | 0.0051 | 0.0690 | 0.0503 | 0.0406 | 0.0118 | 0.0219 | 0.1021 | 0.0453 | 0.0780 | 0.0221 | 0.0535 | 0.0198 | 0.0657 | 0.0101 | 0.0581 | 0.0790 | 0.0499 | 0.0216 | **0.9343** |
| **E_3_** | 0.0167 | 0.0330 | 0.1102 | 0.0059 | 0.0989 | 0.0676 | 0.0586 | 0.0138 | 0.0140 | 0.0694 | 0.0982 | 0.0815 | 0.0738 | 0.0896 | 0.0277 | 0.0914 | 0.0136 | 0.0671 | 0.0915 | 0.0854 | 0.0796 | **1.2875** |
| **E_4_** | 0.0202 | 0.0368 | 0.1238 | 0.0071 | 0.1049 | 0.0695 | 0.0532 | 0.0157 | 0.0163 | 0.1085 | 0.0429 | 0.1093 | 0.0421 | 0.0802 | 0.0877 | 0.1056 | 0.0368 | 0.0678 | 0.1094 | 0.0578 | 0.0779 | **1.3737** |
| **E_5_** | 0.0191 | 0.0364 | 0.1205 | 0.0060 | 0.1015 | 0.0635 | 0.0495 | 0.0160 | 0.0146 | 0.0592 | 0.0874 | 0.0456 | 0.1000 | 0.0979 | 0.0881 | 0.0913 | 0.0362 | 0.0667 | 0.0928 | 0.0856 | 0.0791 | **1.3571** |
| **Ec_1_** | 0.0014 | 0.0071 | 0.0096 | 0.0003 | 0.0501 | 0.0411 | 0.0071 | 0.0021 | 0.0021 | 0.0049 | 0.0028 | 0.0041 | 0.0036 | 0.0743 | 0.0050 | 0.0074 | 0.0019 | 0.0043 | 0.0050 | 0.0063 | 0.0050 | **0.2454** |
| **Ec_2_** | 0.0541 | 0.0930 | 0.1328 | 0.0060 | 0.0992 | 0.0761 | 0.0834 | 0.0200 | 0.0202 | 0.0915 | 0.0561 | 0.0622 | 0.0555 | 0.1250 | 0.0953 | 0.1064 | 0.0622 | 0.0881 | 0.0925 | 0.0347 | 0.0290 | **1.4833** |
| **Ec_3_** | 0.0090 | 0.0762 | 0.0975 | 0.0014 | 0.0879 | 0.0694 | 0.0187 | 0.0111 | 0.0112 | 0.0205 | 0.0135 | 0.0172 | 0.0146 | 0.0925 | 0.0294 | 0.0970 | 0.0098 | 0.0183 | 0.0216 | 0.0191 | 0.0160 | **0.7518** |
| **Ec_4_** | 0.0075 | 0.0194 | 0.0754 | 0.0009 | 0.0799 | 0.0272 | 0.0144 | 0.0059 | 0.0058 | 0.0143 | 0.0089 | 0.0116 | 0.0100 | 0.0872 | 0.0392 | 0.0870 | 0.0809 | 0.0180 | 0.0137 | 0.0121 | 0.0098 | **0.6292** |
| **Ec_5_** | 0.0773 | 0.0238 | 0.0853 | 0.0010 | 0.0835 | 0.0232 | 0.0144 | 0.0104 | 0.0097 | 0.0178 | 0.0115 | 0.0148 | 0.0126 | 0.0920 | 0.0280 | 0.0917 | 0.0077 | 0.0909 | 0.0151 | 0.0134 | 0.0109 | **0.7349** |
| **Ec_6_** | 0.0760 | 0.0655 | 0.1679 | 0.0745 | 0.1506 | 0.1199 | 0.1237 | 0.0800 | 0.0793 | 0.1374 | 0.0995 | 0.1104 | 0.0985 | 0.1566 | 0.1069 | 0.1322 | 0.0267 | 0.1229 | 0.1500 | 0.1246 | 0.0974 | **2.3004** |
| **S_1_** | 0.0150 | 0.0748 | 0.0994 | 0.0051 | 0.0814 | 0.0241 | 0.0572 | 0.0134 | 0.0135 | 0.0282 | 0.0178 | 0.0229 | 0.0186 | 0.0576 | 0.0364 | 0.0299 | 0.0114 | 0.0546 | 0.0782 | 0.0969 | 0.0455 | **0.8819** |
| **S_2_** | 0.0158 | 0.0473 | 0.0843 | 0.0068 | 0.1099 | 0.0965 | 0.0633 | 0.0148 | 0.0154 | 0.1013 | 0.0226 | 0.0866 | 0.0223 | 0.0600 | 0.0246 | 0.0424 | 0.0124 | 0.0649 | 0.1053 | 0.0894 | 0.1009 | **1.1868** |
| **R** | **0.7337** | **1.3653** | **2.3721** | **0.2479** | **1.9779** | **1.4038** | **1.2415** | **0.7359** | **0.7200** | **1.5539** | **1.0950** | **1.3214** | **1.0778** | **2.1228** | **1.0396** | **1.7497** | **0.6212** | **1.3695** | **1.6503** | **1.2928** | **1.0698** | **0.0607** |

**Table S-10** Effect or inner dependence matrix

| **Criteria** | **T_1_** | **T_2_** | **T_3_** | **T_4_** | **T_5_** | **T_6_** | **T_7_** | **T_8_** | **E_1_** | **E_2_** | **E_3_** | **E_4_** | **E_5_** | **Ec_1_** | **Ec_2_** | **Ec_3_** | **Ec_4_** | **Ec_5_** | **Ec_6_** | **S_1_** | **S_2_** |
| --- | --- | --- | --- | --- | --- | --- | --- | --- | --- | --- | --- | --- | --- | --- | --- | --- | --- | --- | --- | --- | --- |
| **T_1_** | 0.0929 | 0.0909 | 0.1211 | 0.0000 | 0.0000 | 0.0000 | 0.0000 | 0.0741 | 0.0649 | 0.0723 | 0.0000 | 0.0625 | 0.0000 | 0.1089 | 0.0000 | 0.1031 | 0.0000 | 0.0982 | 0.0000 | 0.0000 | 0.0000 |
| **T_2_** | 0.0000 | 0.1053 | 0.1196 | 0.0000 | 0.0771 | 0.0627 | 0.0000 | 0.0673 | 0.0705 | 0.0775 | 0.0000 | 0.0668 | 0.0000 | 0.1153 | 0.0000 | 0.0000 | 0.0000 | 0.0712 | 0.1070 | 0.0695 | 0.0626 |
| **T_3_** | 0.0792 | 0.1089 | 0.1611 | 0.0000 | 0.1044 | 0.0743 | 0.0633 | 0.0655 | 0.0637 | 0.1203 | 0.0893 | 0.1027 | 0.0878 | 0.1276 | 0.0000 | 0.1241 | 0.0623 | 0.1057 | 0.1117 | 0.0868 | 0.0759 |
| **T_4_** | 0.0871 | 0.1270 | 0.1745 | 0.0828 | 0.1386 | 0.1048 | 0.1144 | 0.0876 | 0.0855 | 0.1350 | 0.0992 | 0.1110 | 0.0995 | 0.1586 | 0.0946 | 0.1338 | 0.0000 | 0.1145 | 0.1338 | 0.1084 | 0.0812 |
| **T_5_** | 0.0000 | 0.0841 | 0.1124 | 0.0000 | 0.1122 | 0.0000 | 0.0819 | 0.0000 | 0.0000 | 0.0000 | 0.0000 | 0.0000 | 0.0000 | 0.1038 | 0.0000 | 0.0847 | 0.0000 | 0.0000 | 0.0000 | 0.0765 | 0.0000 |
| **T_6_** | 0.0000 | 0.0951 | 0.1288 | 0.0000 | 0.0874 | 0.1138 | 0.0986 | 0.0000 | 0.0000 | 0.0641 | 0.0000 | 0.0000 | 0.0000 | 0.1206 | 0.0000 | 0.1013 | 0.0000 | 0.0835 | 0.0732 | 0.0855 | 0.0746 |
| **T_7_** | 0.0000 | 0.0991 | 0.1268 | 0.0000 | 0.1044 | 0.0814 | 0.1047 | 0.0000 | 0.0000 | 0.0800 | 0.0000 | 0.0000 | 0.0000 | 0.1206 | 0.0000 | 0.1157 | 0.0000 | 0.0686 | 0.0806 | 0.0000 | 0.0000 |
| **T_8_** | 0.0000 | 0.0837 | 0.1185 | 0.0000 | 0.1053 | 0.0820 | 0.0632 | 0.0929 | 0.0000 | 0.0977 | 0.0845 | 0.0844 | 0.0835 | 0.1178 | 0.0776 | 0.0000 | 0.0000 | 0.0000 | 0.1033 | 0.0000 | 0.0000 |
| **E_1_** | 0.0000 | 0.0000 | 0.1098 | 0.0000 | 0.0718 | 0.0000 | 0.0000 | 0.0000 | 0.0893 | 0.0943 | 0.0820 | 0.0880 | 0.0805 | 0.0832 | 0.0767 | 0.0000 | 0.0000 | 0.0000 | 0.0946 | 0.0864 | 0.0000 |
| **E_2_** | 0.0000 | 0.0000 | 0.0928 | 0.0000 | 0.0690 | 0.0000 | 0.0000 | 0.0000 | 0.0000 | 0.1021 | 0.0000 | 0.0780 | 0.0000 | 0.0000 | 0.0000 | 0.0657 | 0.0000 | 0.0000 | 0.0790 | 0.0000 | 0.0000 |
| **E_3_** | 0.0000 | 0.0000 | 0.1102 | 0.0000 | 0.0989 | 0.0676 | 0.0000 | 0.0000 | 0.0000 | 0.0694 | 0.0982 | 0.0815 | 0.0738 | 0.0896 | 0.0000 | 0.0914 | 0.0000 | 0.0671 | 0.0915 | 0.0854 | 0.0796 |
| **E_4_** | 0.0000 | 0.0000 | 0.1238 | 0.0000 | 0.1049 | 0.0695 | 0.0000 | 0.0000 | 0.0000 | 0.1085 | 0.0000 | 0.1093 | 0.0000 | 0.0802 | 0.0877 | 0.1056 | 0.0000 | 0.0678 | 0.1094 | 0.0000 | 0.0779 |
| **E_5_** | 0.0000 | 0.0000 | 0.1205 | 0.0000 | 0.1015 | 0.0635 | 0.0000 | 0.0000 | 0.0000 | 0.0000 | 0.0874 | 0.0000 | 0.1000 | 0.0979 | 0.0881 | 0.0913 | 0.0000 | 0.0667 | 0.0928 | 0.0856 | 0.0791 |
| **Ec_1_** | 0.0000 | 0.0000 | 0.0000 | 0.0000 | 0.0000 | 0.0000 | 0.0000 | 0.0000 | 0.0000 | 0.0000 | 0.0000 | 0.0000 | 0.0000 | 0.0743 | 0.0000 | 0.0000 | 0.0000 | 0.0000 | 0.0000 | 0.0000 | 0.0000 |
| **Ec_2_** | 0.0000 | 0.0930 | 0.1328 | 0.0000 | 0.0992 | 0.0761 | 0.0834 | 0.0000 | 0.0000 | 0.0915 | 0.0000 | 0.0622 | 0.0000 | 0.1250 | 0.0953 | 0.1064 | 0.0622 | 0.0881 | 0.0925 | 0.0000 | 0.0000 |
| **Ec_3_** | 0.0000 | 0.0762 | 0.0975 | 0.0000 | 0.0879 | 0.0694 | 0.0000 | 0.0000 | 0.0000 | 0.0000 | 0.0000 | 0.0000 | 0.0000 | 0.0925 | 0.0000 | 0.0970 | 0.0000 | 0.0000 | 0.0000 | 0.0000 | 0.0000 |
| **Ec_4_** | 0.0000 | 0.0000 | 0.0754 | 0.0000 | 0.0799 | 0.0000 | 0.0000 | 0.0000 | 0.0000 | 0.0000 | 0.0000 | 0.0000 | 0.0000 | 0.0872 | 0.0000 | 0.0870 | 0.0809 | 0.0000 | 0.0000 | 0.0000 | 0.0000 |
| **Ec_5_** | 0.0773 | 0.0000 | 0.0853 | 0.0000 | 0.0835 | 0.0000 | 0.0000 | 0.0000 | 0.0000 | 0.0000 | 0.0000 | 0.0000 | 0.0000 | 0.0920 | 0.0000 | 0.0917 | 0.0000 | 0.0909 | 0.0000 | 0.0000 | 0.0000 |
| **Ec_6_** | 0.0760 | 0.0655 | 0.1679 | 0.0745 | 0.1506 | 0.1199 | 0.1237 | 0.0800 | 0.0793 | 0.1374 | 0.0995 | 0.1104 | 0.0985 | 0.1566 | 0.1069 | 0.1322 | 0.0000 | 0.1229 | 0.1500 | 0.1246 | 0.0974 |
| **S_1_** | 0.0000 | 0.0748 | 0.0994 | 0.0000 | 0.0814 | 0.0000 | 0.0000 | 0.0000 | 0.0000 | 0.0000 | 0.0000 | 0.0000 | 0.0000 | 0.0000 | 0.0000 | 0.0000 | 0.0000 | 0.0000 | 0.0782 | 0.0969 | 0.0000 |
| **S_2_** | 0.0000 | 0.0000 | 0.0843 | 0.0000 | 0.1099 | 0.0965 | 0.0633 | 0.0000 | 0.0000 | 0.1013 | 0.0000 | 0.0866 | 0.0000 | 0.0000 | 0.0000 | 0.0000 | 0.0000 | 0.0649 | 0.1053 | 0.0894 | 0.1009 |

**Table S-11** Degree of relationship

| **Data statistics** | **Value** | Degree of relationship |
| --- | --- | --- |
| Minimum | 0.0622 | No relation < 0.0622 |
| Average | 0.0942 | 0.0622 ≤ Weak ≤ 0.0942 |
| Higher | 0.1344 | 0.0943 ≤ Medium ≤ 0.1344 |
| Max | 0.1745 | Strong > 0.1344 |
